# Viral and Cellular Proteins Containing FGDF Motifs Bind G3BP to Block Stress Granule Formation

**DOI:** 10.1371/journal.ppat.1004659

**Published:** 2015-02-06

**Authors:** Marc D. Panas, Tim Schulte, Bastian Thaa, Tatiana Sandalova, Nancy Kedersha, Adnane Achour, Gerald M. McInerney

**Affiliations:** 1 Department of Microbiology, Tumor and Cell Biology, Karolinska Institutet, Stockholm, Sweden; 2 Science for Life Laboratory, Department of Medicine Solna, Karolinska Institutet, Stockholm, Sweden; 3 Division of Rheumatology, Immunology and Allergy, Brigham and Women's Hospital, Boston, Massachusetts, United States of America; University of North Carolina at Chapel Hill, UNITED STATES

## Abstract

The Ras-GAP SH3 domain–binding proteins (G3BP) are essential regulators of the formation of stress granules (SG), cytosolic aggregates of proteins and RNA that are induced upon cellular stress, such as virus infection. Many viruses, including Semliki Forest virus (SFV), block SG induction by targeting G3BP. In this work, we demonstrate that the G3BP-binding motif of SFV nsP3 consists of two FGDF motifs, in which both phenylalanine and the glycine residue are essential for binding. In addition, we show that binding of the cellular G3BP-binding partner USP10 is also mediated by an FGDF motif. Overexpression of wt USP10, but not a mutant lacking the FGDF-motif, blocks SG assembly. Further, we identified FGDF-mediated G3BP binding site in herpes simplex virus (HSV) protein ICP8, and show that ICP8 binding to G3BP also inhibits SG formation, which is a novel function of HSV ICP8. We present a model of the three-dimensional structure of G3BP bound to an FGDF-containing peptide, likely representing a binding mode shared by many proteins to target G3BP.

## Author Summary

Stress granules (SGs) are dynamic aggregates of proteins and translationally silenced mRNA that are formed in cells upon various stress conditions, such as virus infection. SGs are thought to be antiviral, and many viruses have hence evolved countermeasures to prevent their formation, often targeting the essential SG protein G3BP. Here, we show that several otherwise unrelated viral and cellular proteins all bind G3BP with the sequence motif FGDF, and thereby repress SG formation: the non-structural protein 3 (nsP3) of the Old World alphavirus Semliki Forest virus (a close relative of the emerging, highly pathogenic Chikungunya virus); the protein ICP8 of herpes simplex virus; and in addition, the cellular protein USP10 (an SG component and protein deubiquitinase that stabilises e.g. the tumor suppressor p53). In this work, we also present and validate a model of the three-dimensional structure of G3BP bound to an FGDF-containing peptide. The FGDF-mediated G3BP binding represents an attractive target for therapeutic interventions against a range of diverse viral infections, and may also regulate the p53-stabilising function of USP10 in cancers.

## Introduction

The Ras-GAP SH3 domain–binding proteins (G3BP) are multifunctional RNA-binding proteins, present in two forms, G3BP-1 and G3BP-2 (here collectively referred to as G3BP). They have a well-described importance in mediating the formation of RNA stress granules (SG), both in cells exposed to environmental stress and viral infections [1,2]. SGs are formed when translation initiation is compromised after phosphorylation of eukaryotic initiation factor eIF2α [3] or inhibition of eIF4A [4]. The assembly of SGs allows for rapid redirection of translation to stress response mRNAs or, in the case of viral infection, for inhibition of viral gene expression. The G3BP proteins possess RNA recognition motifs (RRM), which, together with protein/protein interaction domains, are required for SG induction [2]. The N-terminus of G3BP comprises a nuclear transport factor 2 (NTF2)-like domain [5], which is likely involved in dimerization [5,6], but little is known about the functional consequences of such dimerization. The G3BP NTF2-like domain forms complexes with a number of cellular proteins such as ubiquitin-specific protease 10 (USP10), caprin-1 and OGFOD-1 [7–9]. G3BP-binding regulates the activity of USP10, a predominantly cytoplasmic deubiquitinating enzyme (DUB) [8] which stabilizes several important proteins including the cystic fibrosis transmembrane conductance regulator (CFTR) [10], the tumor suppressor p53 [11], the autophagy regulator Beclin-1 [12], the sirtuin family histone deacetylase SIRT6 [13], the NF‐kB essential modulator (NEMO/IKKγ) [14] and the transporter associated with antigen processing (TAP1) [15]. The G3BP binding region of USP10 is found within its N-terminal 76 residues [16], and this interaction inhibits the DUB activity [8,17].

SGs are induced by many virus infections and in turn, viruses have evolved many countermeasures, often targeting G3BP [18]. SG assembly in poliovirus infection is inhibited by cleavage of G3BP between residues Q325 and G326 by the viral 3C protease [19] separating the NTF2-like and RRM domains and leading to the formation of compositionally distinct SGs, lacking G3BP [20]. For some viruses, G3BP is recruited to foci of viral protein accumulation and may be important for efficient completion of the viral life cycle. In vaccinia virus (VV)-infected cells, G3BP is recruited to the cytoplasmic viral factories [21]. However, it has also been reported to have an antiviral role in VV infection [22]. Likewise, G3BP has been implicated as a potential component of the hepatitis C virus (HCV) replication complex [23] and may play an important role in virus assembly [24]. We and others have shown that the G3BP NTF2-like domain is directly bound by L/ITFGDFD repeat motifs in the C-termini of non-structural protein (nsP)3 of the Old World alphaviruses, including Semliki Forest virus (SFV) and chikungunya virus (CHIKV) [25–28]. Subsequent sequestration of G3BP to foci of viral protein accumulation renders the infected cells unable to assemble SGs, despite sustained high levels of eIF2α phosphorylation [28,29].

In this work, we set out to precisely define the characteristics of the G3BP-binding motif in the Old World alphavirus nsP3. We found that the core binding motif consists of two phenylalanine residues separated by a glycine and an aspartate residue (FGDF). Mutation of either of these residues prevented binding to either G3BP-1 or G3BP-2. We also find FGDF motifs at the N-terminus of USP10 and at the C-terminus of herpes simplex virus (HSV)-1 protein ICP8, and confirmed that these motifs similarly bind G3BP and block SG induction. Finally, we generated a molecular model of the G3BP/FGDF peptide interaction and validated the model by site-directed mutagenesis. These results reveal the mechanism by which at least two pathogenic viruses target G3BP. Moreover, they also provide a molecular basis for the regulation of USP10 activity by assembly of the inhibitory USP10/G3BP complex.

## Results

### The G3BP-binding domain in SFV nsP3 contains a core binding motif of FGDF

**Fig 1:**
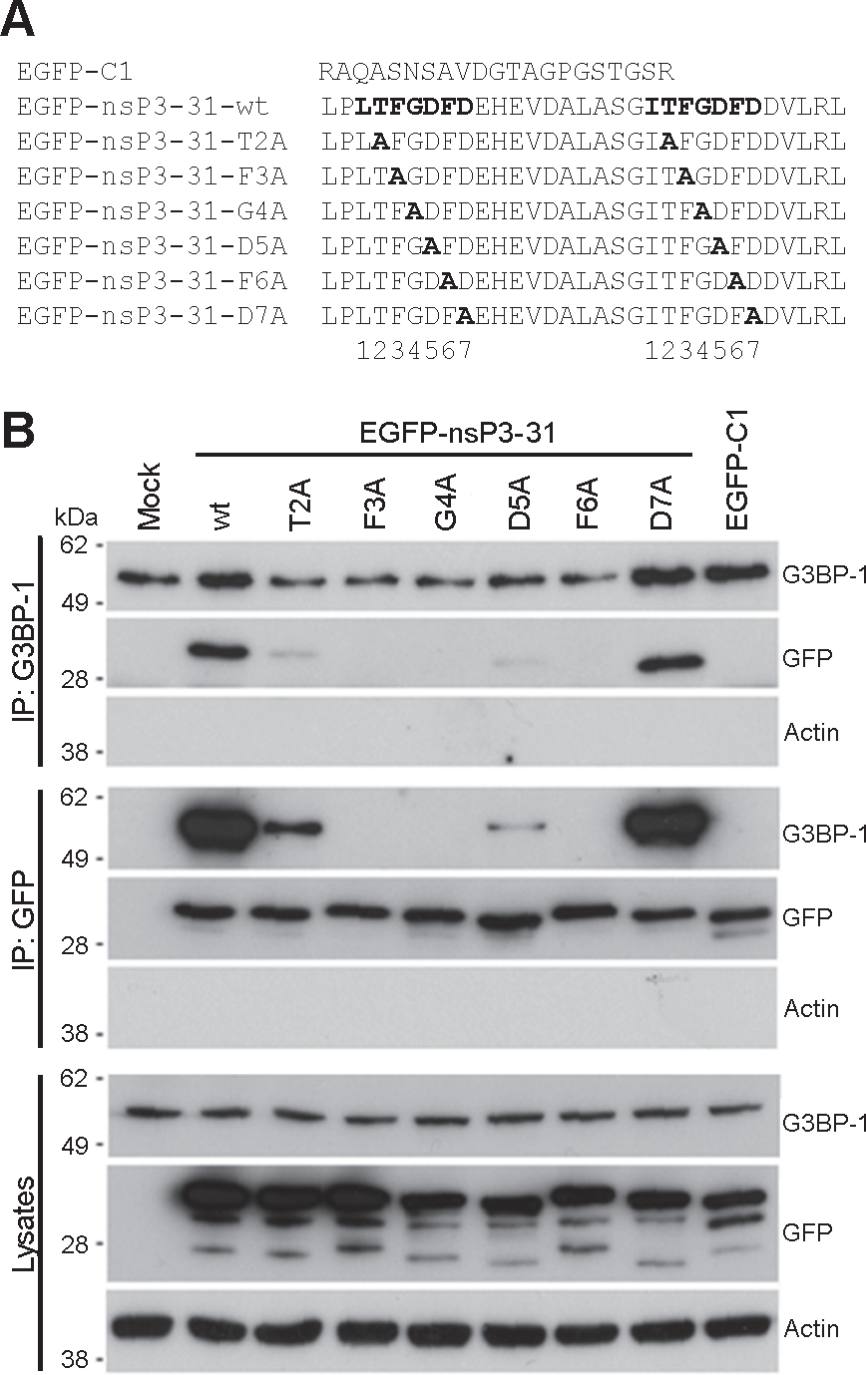
Mutagenesis of the G3BP-binding domain in SFV-nsP3 reveals a core binding motif of FGDF. (A) C-terminal sequences of pEGFP-C1, pEGFP-nsP3-31-wt, -T2A, -F3A, -G4A, -D5A, -F6A or -D7A. Alanine mutations are shown in bold. (B) BHK cells were mock transfected (M) or transfected with pEGFP-nsP3-31-wt, -T2A, -F3A, -G4A, -D5A, -F6A or -D7A or pEGFP-C1. Cell lysates were prepared 16 h after transfection and immunoprecipitated with anti-GFP or G3BP-1 antisera and separated by SDS–PAGE and transferred to nitrocellulose. Blots of total lysates and IPs were probed for G3BP-1, GFP or actin.

The C-terminal L/ITFGDFD repeat motifs, which constitute the G3BP-binding site of nsP3, are well conserved in the Old World alphaviruses [30] with particularly strong conservation of the phenylalanine residues at positions 3 and 6 of the seven residue motif and of the glycine residue at position 4. In order to identify essential residues in the SFV sequence for G3BP binding, we constructed mutants in which each residue from T2 to D7 in both repeats was exchanged for alanine (Fig. 1A). These were constructed in the context of the pEGFP-nsP3-31 construct, containing amino acids 447–477 of SFV nsP3 fused to the C-terminus of EGFP. The empty pEGFP-C1 vector, encoding EGFP with a C-terminal extension of 21 residues was used as a control. We previously demonstrated that EGFP-nsP3-31 and an analogous EGFP fusion protein containing amino acids 475–523 of the CHIKV nsP3 efficiently bind G3BP in transient transfection experiments [27,28]. BHK cells were transfected with vectors encoding EGFP alone, EGFP-nsP3-31 or each of the alanine mutants. Lysates were immunoprecipitated with G3BP-1 antibody and immunoblotted with antibodies against G3BP-1, GFP or actin (Fig. 1B, upper panels). As expected, EGFP-nsP3-31, containing the wild type (wt) nsP3 sequence, efficiently bound G3BP-1, but EGFP alone did not. The construct carrying a mutation of the threonine residue T2 to an alanine exhibited weak but readily detectable binding to G3BP. However, mutation of either of the residues F3, G4 or F6 completely disrupted binding. Finally, while mutation of the aspartate residues at D5 to alanine substantially reduced binding, mutation of D7 had no effect. Lysates were also immunoprecipitated with anti-GFP and immunoblotted for G3BP-1, GFP or actin (Fig. 1B, middle panels), with similar results. Furthermore, GFP-immunoprecipitates were immunoblotted for G2BP-2 (S1 Fig.). These results show that both alternatively-spliced isoforms of G3BP-2 (a and b) interact with EGFP-31 constructs in a similar manner to G3BP-1. We conclude from these results that residues F3, G4 and F6 are essential for G3BP-binding and that T2 and D5 contribute significantly.

### SFV-F3A does not sequester G3BP, induces a stronger SG response and is attenuated in tissue culture

**Fig 2:**
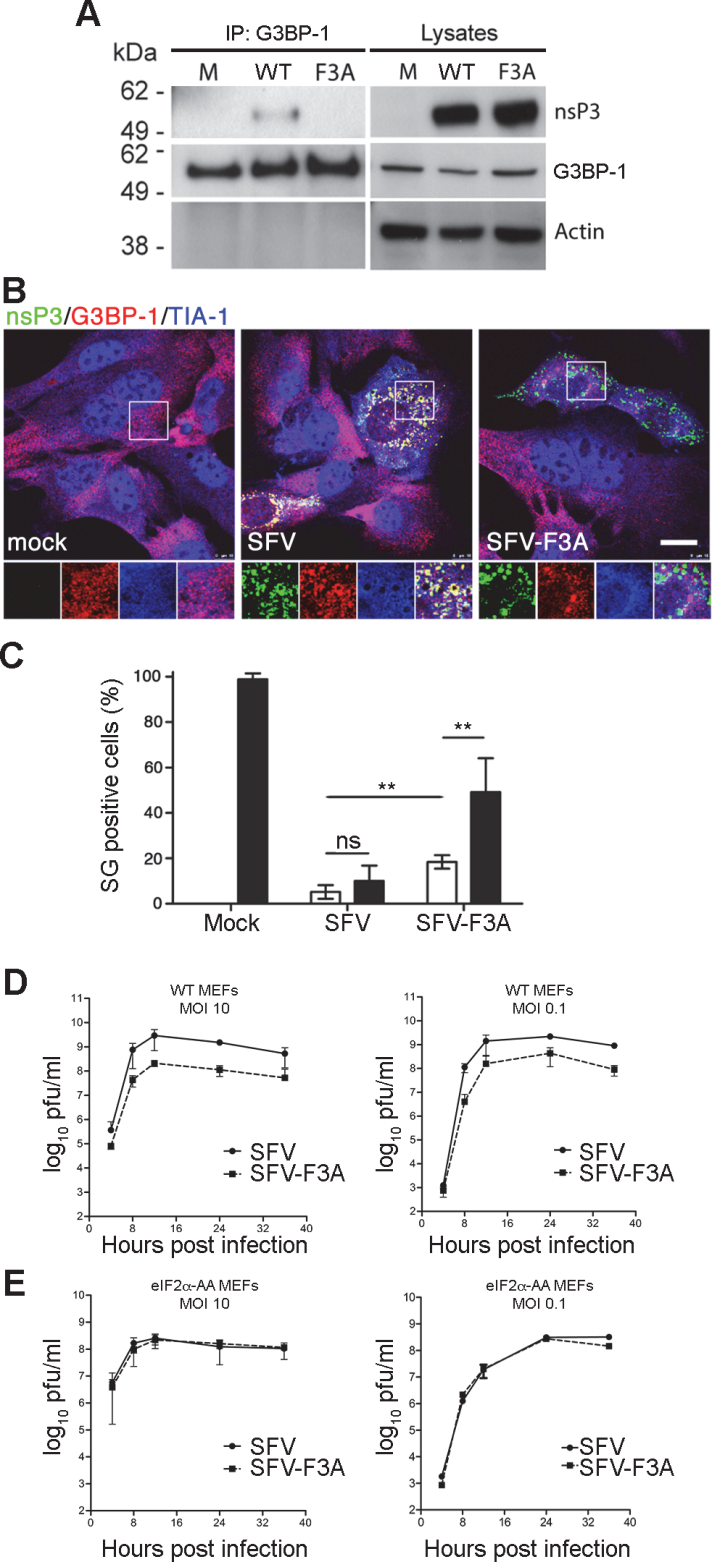
SFV-F3A does not sequester G3BP, induces a stronger SG response and is attenuated *in vitro*. (A) BHK cells were infected at MOI 10 with SFV-wt or SFV-F3A. At 8 hpi, cell lysates were prepared and immunoprecipitated with G3BP-1 antisera and separated by SDS–PAGE. Lysates and IPs were probed for nsP3, G3BP-1 or actin. (B) MEF cells were infected with SFV-wt or SFV-F3A at MOI 1. At 8 hpi cells were fixed and stained for nsP3 (green), G3BP-1 (red) and TIA-1 (blue). Bar 20 μm. (C) MEF cells were mock infected or infected with SFV-wt or SFV-F3A at an MOI of 50 for 7 h before 1h treatment with Pat A (100 nM) or mock treatment. Cells were fixed and stained for G3BP-1, TIA-1 and nsP3. Fifty cells per treatment were scored as SG+ or not based on G3BP-1 and TIA-1 colocalization. Data are presented as mean ± SD from five independent experiments. Open bars, mock treated; closed bars, Pat A. Unpaired Student’s t test, **p < 0.01. (D) WT or (E) eIF2α-AA MEFs were infected with SFV-wt or SFV-F3A at an MOI of 10 (left) or an MOI of 0.1 (right). At 4, 8, 12, 24 and 36 hpi, supernatants were collected, and SFV titers were quantified by plaque assay on BHK cells. Data are means of two independent experiments. Error bars indicate SD.

Previously we have shown that in Old World alphavirus-infected cells, the C-terminal repeat sequences of nsP3 bind and sequester both G3BP-1 and G3BP-2 into foci of viral protein accumulation including cytopathic vacuoles (CPV) [27,28,31]. In order to test the effects of the non-G3BP-binding mutations on the life cycle of SFV, we mutated both nsP3 residues F451 and F468 to alanines in the infectious clone of SFV (corresponding to the mutation F3A in the two (L/I)TFGDFD motifs, hereafter referred to as SFV-F3A). Previously, we showed that SFV-Δ789, lacking nsP3 residues 449–472, including the G3BP-binding sites displayed slower processing of the P34 precursor [28]. To determine if SFV-F3A also displays slow processing phenotype, we infected BHK cells with wt SFV, SFV-F3A or SFV-Δ789 and compared levels of P34. Lysates from WT SFV or SFV-F3A-infected cells did not contain detectable levels of P34 (S2A Fig.), and we concluded that the F3A mutations do not result in defective P34 processing. To demonstrate that nsP3-F3A does not bind G3BP-1 in the context of a viral infection, we infected BHK cells with wt SFV or SFV-F3A at MOI 10. Lysates were immunoprecipitated with G3BP-1 antibody and analyzed by immunoblotting for nsP3, G3BP-1 and actin. As expected, nsP3 from wt SFV-infected cells coprecipitated with G3BP-1, but nsP3 F3A did not (Fig. 2A). To determine whether SFV-F3A nsP3 colocalized with G3BP, cells were infected at low MOI with wt SFV or SFV-F3A. Cells were fixed and stained for SFV nsP3, G3BP-1 and TIA-1, another SG marker. As expected, wt SFV nsP3 colocalized well with G3BP-1, with very little colocalization of G3BP-1 with TIA-1 (Fig. 2B). However, nsP3 in SFV-F3A infected cells did not colocalize with G3BP-1, confirming immunoprecipitation data indicating that nsP3 F3A does not interact with G3BP-1. Instead, SFV-F3A infected cells often contained foci of G3BP-1 and TIA-1 co-staining, suggesting longer persistence of SGs in cells infected with the SFV-F3A mutant.

Previously we demonstrated that at late times in SFV infection, when most of the infection-induced SG have been disassembled, SGs cannot be re-induced with phospho-eIF2α- or eIF4A-dependent stress inducers (sodium arsenite or pateamine A (Pat A), respectively) because G3BP is sequestered by nsP3 [28,29]. We next tested whether cells infected with wt SFV or SFV-F3A could mount an SG response to an exogenous stress inducer. Cells were infected with wt SFV or SFV-F3A and stressed with Pat A at 7 hpi for 1 h before fixation and staining for TIA-1 to detect SGs and nsP3 to detect SFV infection (Fig. 2C). Pat A was used since sodium arsenite has little effect in SFV-infected cells as these already display sustained eIF2α phosphorylation [28]. Approximately 100% of mock-infected cells responded to Pat A by forming SGs. After infection with wt SFV, 5.2% of cells had SGs, and this number was not significantly altered by Pat A treatment, confirming that wt SFV–infected cells cannot mount a stress response to secondary stress signals. SFV-F3A–infected cells had a significantly higher number of SG-positive cells than wt SFV–infected cultures (18.4%), and this proportion was increased to 49.2% by the Pat A treatment, indicating that in the absence of nsP3/G3BP interaction, infection-induced SGs persist longer and additional SGs can be stress-induced in SFV-F3A-infected cells. To determine whether the specific ablation of G3BP recruitment affects viral replication, we performed single-step and multi-step growth curves comparing wt SFV and SFV-F3A in MEFs. These data revealed that SFV-F3A propagated to titers between 1.5 and 2 orders of magnitude lower that wt SFV after both low and high MOI infection (Fig. 2D) indicating that blocking the nsP3/G3BP interaction by point mutation of the N-terminal phenylalanine residue in both FGDF motifs attenuates viral infection. Single and multi-step growth curve experiments were also performed in BHK cells with similar results (S2B Fig.). Finally, to address whether the F3A mutations in SFV-nsP3 affect other functions than SG inhibition by G3BP sequestration, we performed viral growth curves in eIF2α-AA MEF cells [32]. These cells contain a mutation at the phosphorylation site (S51A) in eIF2α gene, such that eIF2α cannot be phosphorylated and SGs are not induced. SFV-F3A replication was very similar to WT SFV in those cells (Fig. 2E). This result strongly suggests that the attenuation of SFV-F3A in WT cells is due to the action of a G3BP-dependent process downstream of eIF2α phosphorylation, most likely SG formation. Hence, inhibition of SG formation (in response to infection-induced eIF2α phosphorylation) is probably the primary function of the FGDF motifs in Old World alphavirus nsP3.

### USP10 binds G3BP via a conserved FGDF motif

**Fig 3:**
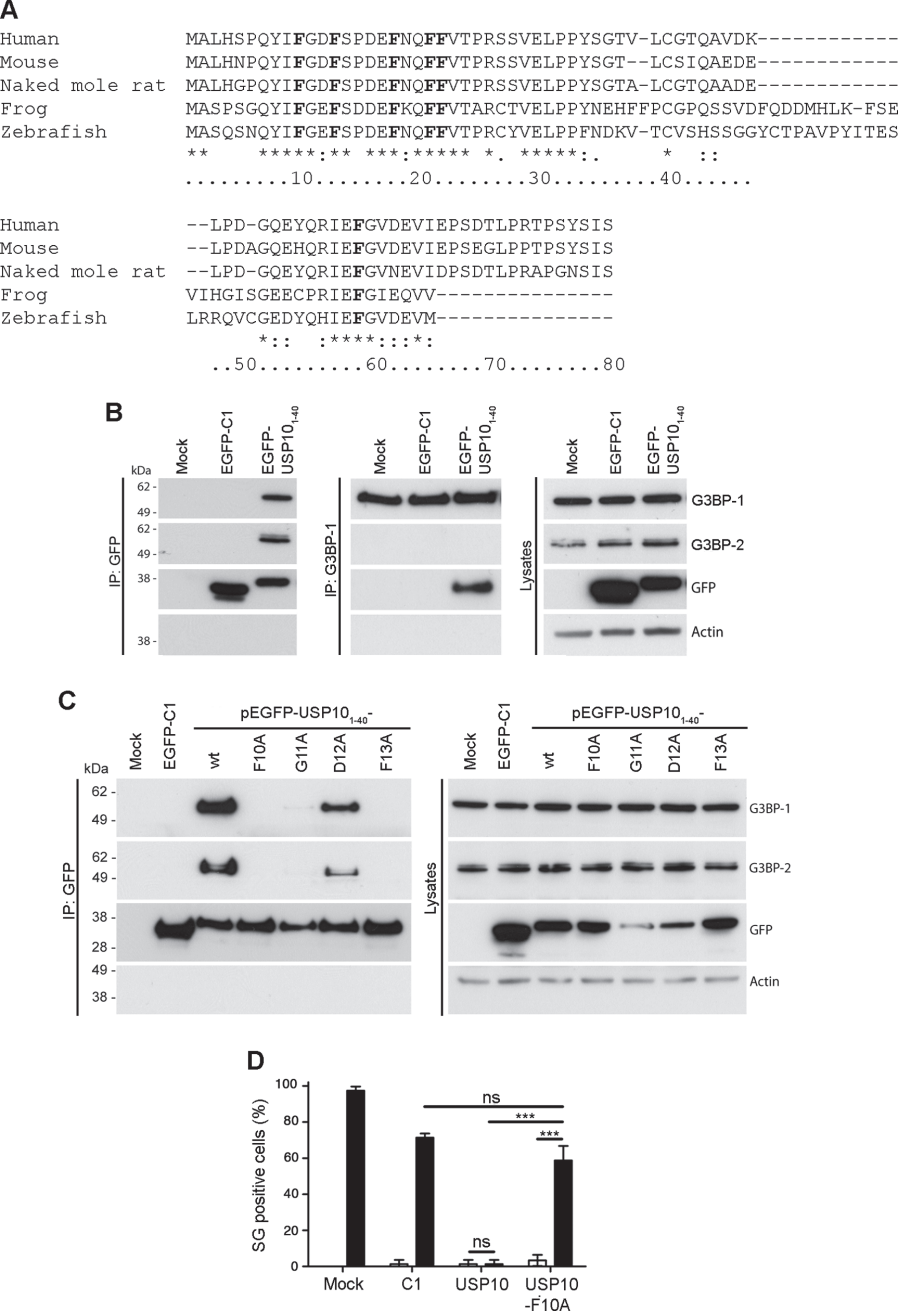
USP10 binds G3BP via a conserved FGDF motif. (A) Alignment of the N-terminal 80 amino acid residues of higher eukaryote USP10. Human (NM_005153.2), mouse (NP_033488.1), naked mole rat (XP_004842781.1), frog (NP_001080643.1), zebrafish (XP_685621.5). The numbering is based on the human sequence. Conserved phenylalanine residues are indicated in bold. (B) BHK cells were mock-transfected or transfected with pEGFP-C1 or pEGFP- USP10_1–40_-wt. Cell lysates were prepared 16 h after transfection and immunoprecipitated with anti-GFP (left panels) or anti-G3BP-1 (middle panels) antisera. Lysates and IPs were separated by SDS-PAGE and probed for G3BP-1, G3BP-2, GFP or actin. (C) BHK cells were mock-transfected (M) or transfected with pEGFP-C1 or pEGFP-USP10_1–40_-wt, -F10A, -G11A, -D12A or -F13A. Cell lysates were prepared 16 h after transfection and immunoprecipitated with anti-GFP and separated by SDS–PAGE. Lysates and IPs were probed for G3BP-1, G3BP-2, GFP or actin. (D) BHK cells were mock-transfected or transfected with pEGFP-C1, pEGFP-USP10-wt or -F10A. After 23 h, the transfected cells were mock-treated or treated with 0.5 mM sodium arsenite for 1 h, fixed and stained for G3BP-1 and TIA1. Fifty cells per treatment were scored as SG+ based on G3BP-1 and TIA-1 colocalization. Open bars, mock treated; closed bars, sodium arsenite. Data are presented as mean ± SD from three independent experiments. Unpaired Student’s t-test, * p < 0.05, *** p < 0.001.

We have previously demonstrated that at late times in infection with SFV, a large proportion of cellular G3BP is recruited to sites of viral protein accumulation [28]. We wondered whether this interaction displaces USP10, a cellular binding partner of G3BP that also binds to the NTF2-like domain [8,25]. When we determined the localization of USP10 in SFV-infected MEFs, we did not detect any significant overlap with G3BP (S3 Fig.). This suggests that USP10 is excluded from the complex with G3BP by the nsP3 interaction, possibly by competition for the same site on G3BP. Recently, the G3BP-binding domain of USP10 was shown to be located within the N-terminal 76 amino acid residues [16]. Having shown that the G3BP-binding motif of nsP3 consists of two FGDF motifs, we asked whether a similar motif exists in the G3BP-binding region of USP10. When we aligned the sequences of the N-terminal 80 residues of USP10 from several species (Fig. 3A), we identified several well conserved phenylalanine residues (F10, F13, F18, F21, F22 and F59 in the human sequence), as well as a conserved FG(D/E)F motif at residues 10–13. We observed particularly high species conservation in the N-terminal 40 residues. To determine whether this USP10 region binds G3BP, we fused the N-terminal 40 residues of human USP10 to EGFP (EGFP-USP10_1–40_). BHK cells were transfected with vectors encoding EGFP alone or EGFP-USP10_1–40_. Lysates were immunoprecipitated with anti-GFP and immunoblotted with sera to G3BP-1, G3BP-2, GFP and actin (Fig. 3B, left panels). Indeed, both G3BP proteins efficiently coprecipitated with EGFP-USP10_1–40_ but not with EGFP alone. Lysates were also immunoprecipitated with G3BP-1 antibody to verify reciprocal coimmunoprecipitation of EGFP-USP10_1–40_ (Fig. 3B, middle panels). To determine whether this binding is dependent on the FGDF motif at residues 10–13, a panel of mutated EGFP-USP10_1–40_ variants was created in which each residue from F10 to F13 was substituted to alanine. Cells were transfected with vectors encoding wt EGFP, EGFP-USP10_1–40_ or each variant. Lysates were immunoprecipitated with anti-GFP and immunoblotted with sera to G3BP-1, G3BP-2, GFP and actin (Fig. 3C). Alanine substitution of F10, G11 or F13 completely disrupted binding, while the D12A mutation allowed weak binding to both G3BP-1 and G3BP-2. Similar results were obtained after G3BP-1 immunoprecipitation (S4 Fig.). These results are remarkably similar to those obtained following mutagenesis of the G3BP-binding domain of SFV nsP3 (Fig. 1), suggesting that both proteins indeed bind G3BP via their FGDF-motifs.

To test if the overexpression and binding of full-length USP10 to G3BP affects the SG response, BHK cells were transfected with EGFP alone, EGFP-USP10 wt or EGFP-USP10-F10A. Transfected cells were mock stressed or stressed with sodium arsenite for 1 h before fixation and staining for G3BP-1 and TIA-1 to detect SGs. Approximately 97% of the mock transfected cells and 71% of the EGFP transfected cells had SGs upon sodium arsenite treatment (Fig. 3D). Overexpression of wt EGFP-USP10 efficiently blocked the ability of the cells to form SGs (just 1% of cells showed SGs), whereas 59% cells transfected with the G3BP-nonbinding mutant EGFP-USP10-F10A responded to sodium arsenite stress with SGs. Representative images are presented in S5 Fig.. Taken together, the results in Fig. 3 show that USP10 acts as a negative regulator of G3BP-dependent SG formation and that this regulation is dependent on G3BP binding by the FGDF motif at residues 10–13.

### SFV nsP3, containing two FGDF repeats, binds 2 molecules of G3BP, while USP10 binds one

**Fig 4:**
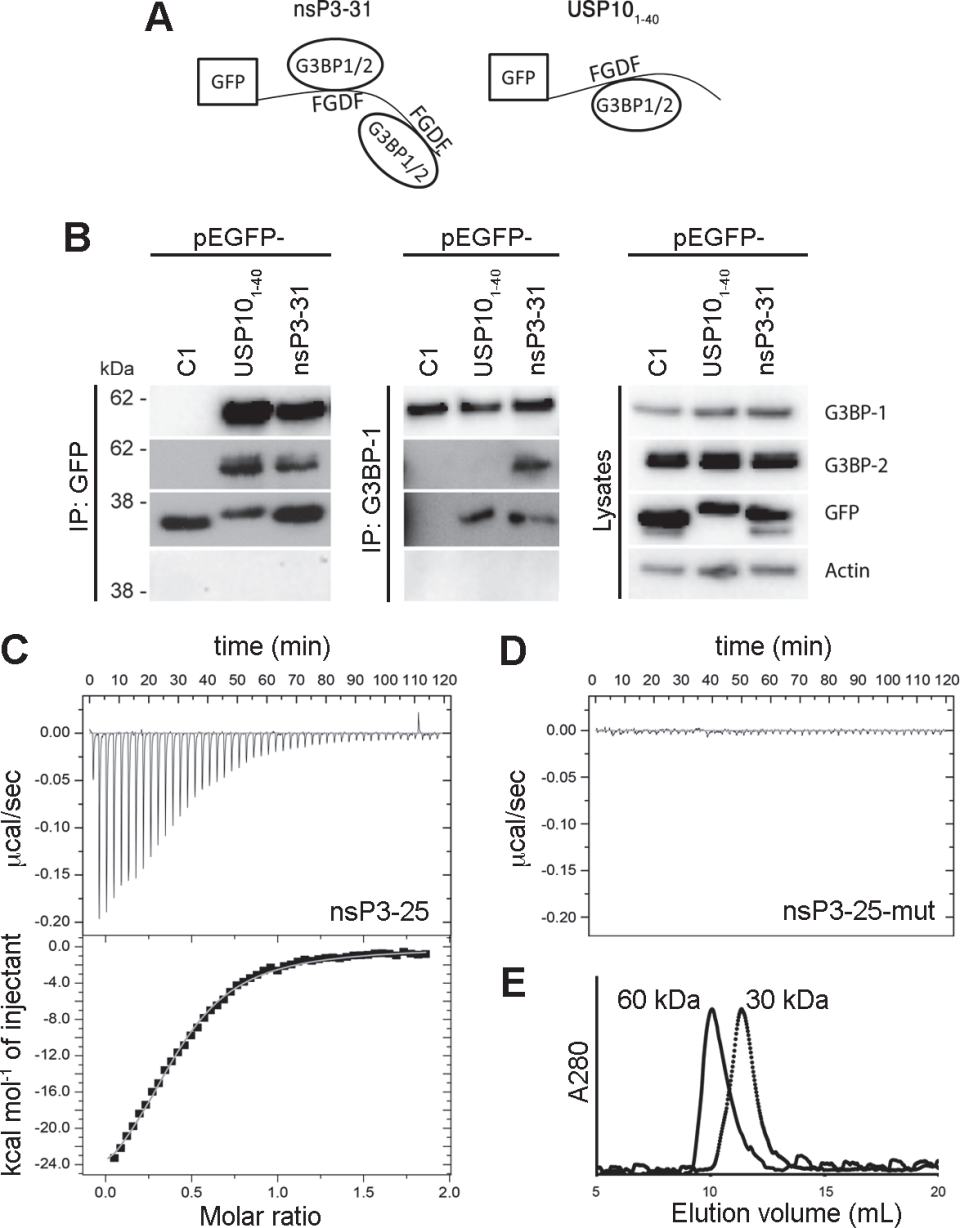
SFV nsP3, containing two FGDF repeats, binds two G3BP molecules, while USP10 binds only one. (A) Schematic representation of the possible binding of the two FGDF motifs in nsP3-31 (left panel) to two G3BP molecules. The right panel shows the possible binding of one G3BP molecule to one FGDF domain of USP10_1–40_. (B) BHK cells were transfected with pEGFP-C1, pEGFP-USP10_1–40_-wt or pEGFP-nsP3-31-wt. Cell lysates were prepared 16 h after transfection and immunoprecipitated with GFP or G3BP-1 antibody and separated by SDS–PAGE. Lysates and IPs were probed for G3BP-1, G3BP-2, GFP or actin. (C) In isothermal titration calorimetry, injection of nsP3-25 (LT**FGDF**DEHEVDALASGIT**FGDF**DD) (syringe: 225 μM) to G3BP-NTF2 (cell: 23 μM) resulted in peptide-concentration dependent exothermic heat changes. The binding isotherm of the integrated heat changes reached half-maximum at a molar ratio of approximately 0.5 demonstrating that two molecules of G3BP-NTF2 were bound by one peptide molecule. A one-to-one binding model was used to fit the binding isotherm and gave the following parameters: number of binding sites (n) on nsP3-25 of 2.4 ± 0.1; association constant (K_A_) of (1.49 ± 0.08)·10^5^ nsP3-25 (dissociation binding constant K_D_ of 6.7 μM); binding enthalpy ΔH of –12.8 ± 0.1 kcal/mol. (D) Titration of the peptide nsP3-25-mut (LT**AGDA**DEHEVDALASGIT**AGDA**DD) to G3BP-NTF2 did not produce binding-induced heat changes. (E) In size exclusion chromatography, G3BP-NTF2 alone (dotted line) or pre-incubated with a ten-fold molar excess of nsP3-25 (solid line) eluted at retention volumes of 11.3 and 10 mL, respectively. This shift corresponded to an increase in the apparent molecular weight from 30 to 60 kDa when G3BP was pre-incubated with nsP3-25. The theoretical MW for G3BP-NTF2 and nsP3-25 are 18.4 kDa and 2.7 kDa, respectively.

In contrast to USP10, nsP3 contains two FGDF motifs (residues 451–454 and 468–471). We therefore hypothesized that a minimal G3BP-binding motif could consist of only one FGDF motif flanked by surrounding residues, which would provide enough space between the two motifs to allow nsP3 to bind two G3BP molecules. Based on our finding that both G3BP-1 and G3BP-2 bind FGDF motifs, we further hypothesized that if EGFP-nsP3-31 binds two molecules of G3BP, it would bind any combination of G3BP-1 and -2, with frequencies depending on the relative abundance of the two proteins and potential differences in affinity. Therefore, G3BP-2 should be detectable in nsP3 complexes immunoprecipitated with anti-G3BP-1 and vice versa, but USP10 should form a complex with only one molecule of either G3BP-1 or -2 (Fig. 4A). To test this, BHK cells were transfected with vectors encoding EGFP, EGFP-USP10_1–40_ or EGFP-nsP3-31. When lysates were immunoprecipitated with anti-GFP, both G3BP-1 and G3BP-2 were detected in complex with EGFP-nsP3-31 and EGFP-USP10_1–40_ (Fig. 4B, left panels). However, when G3BP-1 was immunoprecipitated, G3BP-2 could be detected in EGFP-nsP3-31, but not in EGFP-USP10_1–40_ bound complexes (Fig. 4B, middle panels). This suggests that the nsP3 sequence, containing two FGDF motifs, can form a ternary complex with both G3BP-1 and G3BP-2 simultaneously, while the USP10 sequence, containing only one FGDF motif, can bind either G3BP-1 or G3BP-2, but not both.

Next, we endeavored to confirm the 2:1 stoichiometry of nsP3:G3BP using biophysical *in vitro* measurements. We employed isothermal titration calorimetry (ITC) to determine the affinity, stoichiometry and thermodynamic signature of the interaction between the purified NTF2-like domain of human G3BP-1, expressed in *E*. *coli* (G3BP-NTF2), and a peptide spanning residues 449–473 of SFV nsP3 (nsP3-25), containing two FGDF motifs. Injection of nsP3-25 into a G3BP-NTF2 protein solution resulted in concentration-dependent exothermic heat changes (Fig. 4C, upper panel). The binding isotherm of the integrated heat changes was fitted using a simple one-to-one binding model, yielding an affinity value of 7 μM as well as 2.4 G3BP binding sites per nsP3-25 peptide molecule (Fig. 4C, lower panel). This indicates that one peptide efficiently binds two G3BP molecules. The interaction affinity was 16-fold stronger than the previously determined affinity value of 115 μM for the interaction between a similar G3BP-NTF2 fragment and a DSGFSFGSK peptide [33]. Injection of a control peptide in which both FGDF motifs were exchanged for AGDA (nsP3-25-mut) only produced injection-related heat fluctuations at baseline level (Fig. 4D).

Engagement of two G3BP molecules with each nsP3-25 peptide was further supported by analytical size exclusion chromatography (SEC) analysis. It is known from crystal structures and SEC analysis that the NTF2-like domains of rat and human G3BP form homo-dimers [33]. G3BP-NTF2 alone was eluted with an apparent molecular weight of 30 kDa, slightly lower than the calculated molecular weight of the dimer (36 kDa), while pre-incubation of G3BP with a ten-fold molar excess of nsP3-25 peptide shifted the elution peak, corresponding to the formation of a 60 kDa complex (Fig. 4E). Taking into account the 2:1 ratio for the G3BP-NTF2:nsP3-25 interaction as derived from ITC (Fig. 4C), we suggest that the formed 60 kDa complex comprised four G3BP-NTF2 molecules (two dimers) and two nsP3-25 peptides. There was no evidence for higher-order oligomers.

### The glycine residue of the FGDF motif allows for adequate positioning of both phenylalanine residues into a hydrophobic groove within the G3BP NTF2-like domain

The crystal structures of the NTF2-like domain of G3BP-1 in its apo form and complexed with the nucleoporin (Nup)-derived peptide DSGFSFGSK have recently been determined [33], revealing that the ligand is bound in an extended conformation within a long and deep groove on the surface of G3BP (S6A Fig.). The peptide-binding site is amphipathic; while the base of the groove is highly hydrophobic, both walls lining the cleft of the protein and the positively charged N-terminus are polar with several basic residues (R32, K5, K123). Both phenylalanine residues of the peptide protrude towards well-defined pockets within the hydrophobic groove. While the side chain of residue F4 of the Nup-derived peptide is buried in a deep pocket formed by the G3BP-1 residues F15, F33 and F124, the side chain of residue F6 is localized in a shallower pocket formed by the G3BP-1 residues F124, V11 and L10 [33].

**Fig 5:**
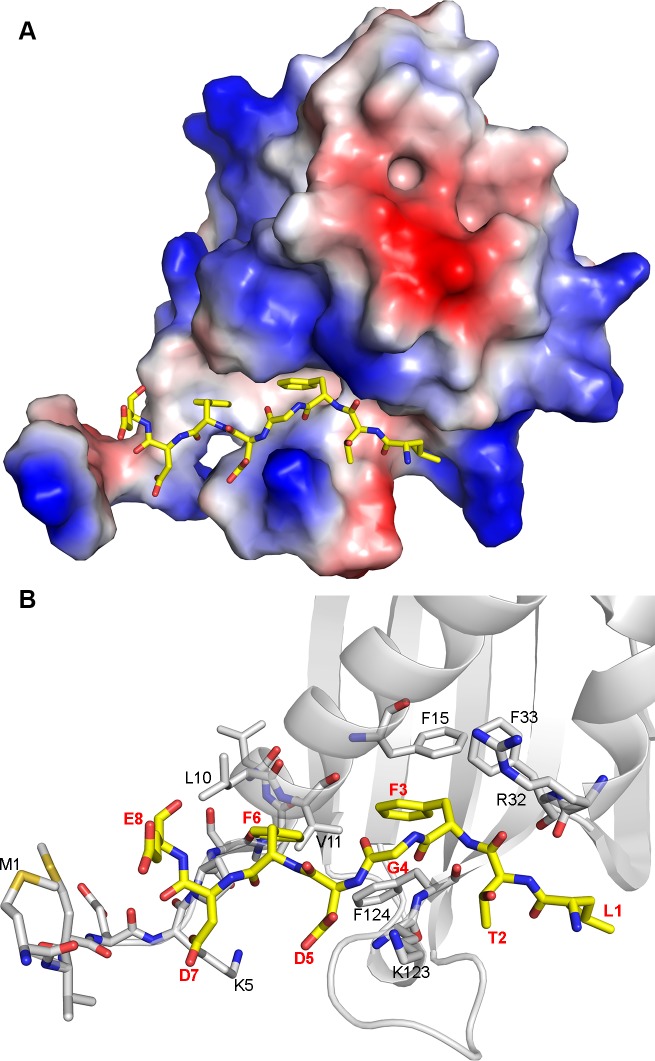
Molecular model of G3BP/FGDF interaction. (A) G3BP-NTF2 (PDB ID: 4FCM) with modelled peptide LTFGDFDE (yellow sticks). The protein is shown as the surface colored according to the electrostatic potential (red: acidic, blue: basic). (B) Details of the interaction of the peptide with G3BP-NTF2. G3BP-NTF2 is shown in the cartoon representation. The N-terminal 11 residues as well as several residues lining the peptide-binding groove are displayed as grey sticks. The LTFGDFDE peptide is displayed as yellow sticks, peptide amino acid residues are labeled in red and in bold font. G3BP-1 residues mentioned in the text are labeled in black.

Our binding and mutagenesis studies reveal that G3BP-1 binds approximately 16 times stronger to FGDF-containing peptides compared to the previously described interaction with the DSGFSFGSK peptide. Both these sequences contain two crucial phenylalanines, which we hypothesized to be bound similarly in both peptides. We assessed this by creating a molecular model in which the octapeptide LTFGDFDE was manually docked into the peptide-binding groove of G3BP-1, using the crystal structure of the G3BP-NTF2 complexed with the DSGFSFGSK peptide as a template (Figs. 5 and S6B). The conformational flexibility of the additional glycine residue within the FGDF motif would allow the phenylalanine side-chains to take similar orientations as in the SGFSF-peptide. Furthermore, the molecular model indicates that the negatively charged residues D5, D7 and E8 in LTFGDFDE could interact with the positively charged side chains of residues K123 and K5 as well as with the positively charged N-terminal region of G3BP (Fig. 5B). It should be noted that these two lysine residues are conserved in human, mouse and *Xenopus* G3BP-1 and are also present in human G3BP-2 (S7 Fig.). The binding mode suggested by the molecular model of the LTFGDFDE/G3BP-1 complex is in agreement with our biochemical analyses, which showed that mutation of the conserved phenylalanine residues at positions 451, 454, 468 and 471 of nsP3 and 10 and 13 of USP10 eliminated G3BP-binding (Figs. 1 and 3). The model might also explain the elimination of binding of both nsP3 and USP10 to G3BP upon mutation of the glycine residue to alanine, which would restrict flexibility and thus hinder adequate positioning of the phenylalanine side-chains in the FGDF motifs.

### Site-directed mutagenesis in the FGDF peptide binding cleft of G3BP

**Fig 6:**
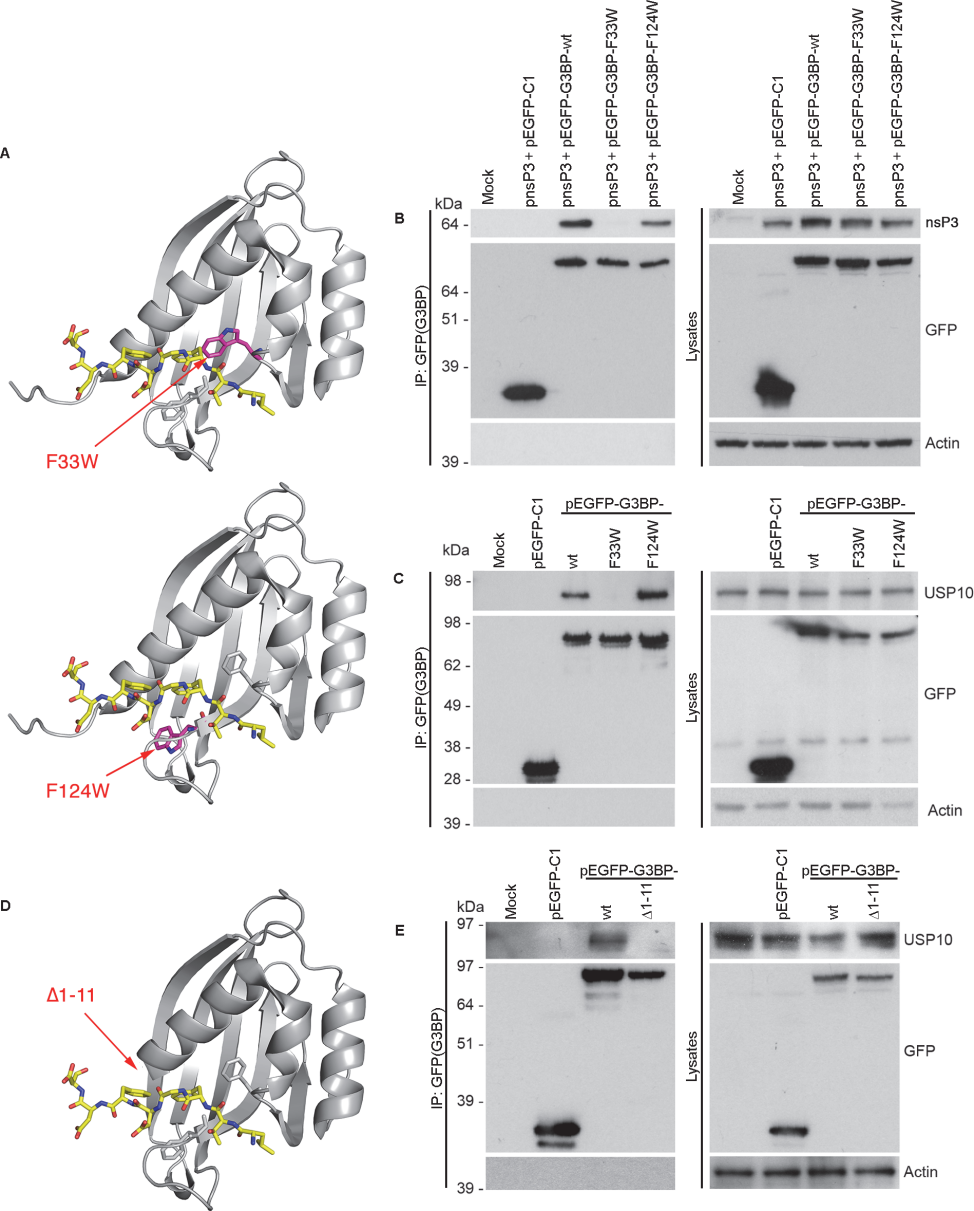
Site-directed mutagenesis in the FGDF peptide binding cleft of G3BP. (A) Schematic representation of the G3BP-F33W mutant (upper panel) and the G3BP-F124W mutant (lower panel) with the modeled LTFGDFDE peptide. The LTFGDFDE peptide is displayed as sticks with yellow carbon atoms. Mutated tryptophan residues are shown in magenta. (B) HEK293T cells were mock transfected or cotransfected with pEBB/PP-SFV-nsP3 (pnsP3) and either pEGFP-C1, pEGFP-G3BP-wt, -F33W, or -F124W. Cell lysates were prepared 24 h after transfection, immunoprecipitated with anti-GFP and separated by SDS–PAGE. Lysates and IPs were probed using streptavidin-HRP (nsP3), GFP or actin. (C) HEK293T cells were mock transfected or transfected with pEGFP-C1, pEGFP-G3BP-wt, -F33W or -F124W. Cell lysates were prepared 24 h after transfection and immunoprecipitated with GFP antisera and separated by SDS–PAGE. Lysates and IPs were probed for USP10, GFP or actin. (D) Schematic representation of G3BP1-Δ1–11 with the modeled LTFGDFDE peptide. The LTFGDFDE peptide is displayed as sticks with yellow carbon atoms. (E) HEK293T cells were mock transfected or transfected with pEGFP-C1, pEGFP-G3BP1-wt or -Δ1–11. Cell lysates were prepared 24 h after transfection and immunoprecipitated with anti-GFP and separated by SDS–PAGE. Lysates and IPs were probed for USP10, GFP or actin.

In order to validate the proposed binding model, residue F33, localized within the hydrophobic pocket of G3BP and predicted to be proximal to residue F3 of the bound LTFGDFDE peptide, was mutated to tryptophan (G3BP-F33W). Residue F33 is buried at the bottom of the peptide-binding cleft, and we hypothesized that its substitution to tryptophan would reduce the size of the pocket, thus hindering adequate positioning of the benzene ring of peptide residue F3 into the cleft (Fig. 6A, upper panel). As a control, residue F124 was also mutated to tryptophan, since this phenylalanine residue is solvent-accessible and not localized within the binding groove (Fig. 6A, lower panel). Notably, F33 is conserved in human, mouse, *Xenopus* and *Aedes* mosquito G3BP-1, while F124 conserved in human, mouse and *Xenopus*, but a tyrosine residue in the *Aedes* sequence (S7A Fig.). Both are conserved in human G3BP-2 (S7B Fig.). To determine whether mutation of these residues affects binding of G3BP with FGDF motifs, nsP3 (tagged with a biotin acceptor peptide, BAP) was co-expressed with either pEGFP-C1, pEGFP-G3BP-wt, pEGFP-G3BP-F33W or pEGFP-G3BP-F124W and binding was analyzed by immunoprecipitation with anti-GFP (Fig. 6B). Consistent with our structural model, EGFP-G3BP-wt and the F124W variant efficiently bound to nsP3-BAP, while EGFP-G3BP-F33W did not detectably interact. Similarly to results with cotransfected nsP3, endogenous USP10 was also coimmunoprecipitated with EGFP-G3BP-wt and EGFP-G3BP-F124W but not with EGFP-G3BP-F33W (Fig. 6C).

The extreme N-terminus of G3BP (residues 1–11) forms a large part of the hydrophobic pocket for positioning of the peptide residue F6 and also contains a lysine residue (K5), possibly interacting with the negative charges of acidic residues downstream of the FGDF motif. The model predicts that a truncated version of G3BP (Δ1–11) would not be capable of binding the peptide (Fig. 6D). To evaluate this, 293T cells were transfected with pEGFP-C1, pEGFP-G3BP-wt or pEGFP-G3BP-∆1–11 and analyzed by immunoprecipitation with anti-GFP and immunoblotting for USP10, GFP or actin (Fig. 6E). Endogenous USP10 coprecipitated with EGFP-G3BP-wt but not with EGFP-G3BP-∆1–11. Taken together, the results in Fig. 6 strongly support our structural model of the G3BP/FGDF complex.

### An FGDF motif in HSV-1 ICP8 binds G3BP and inhibits SGs

**Fig 7:**
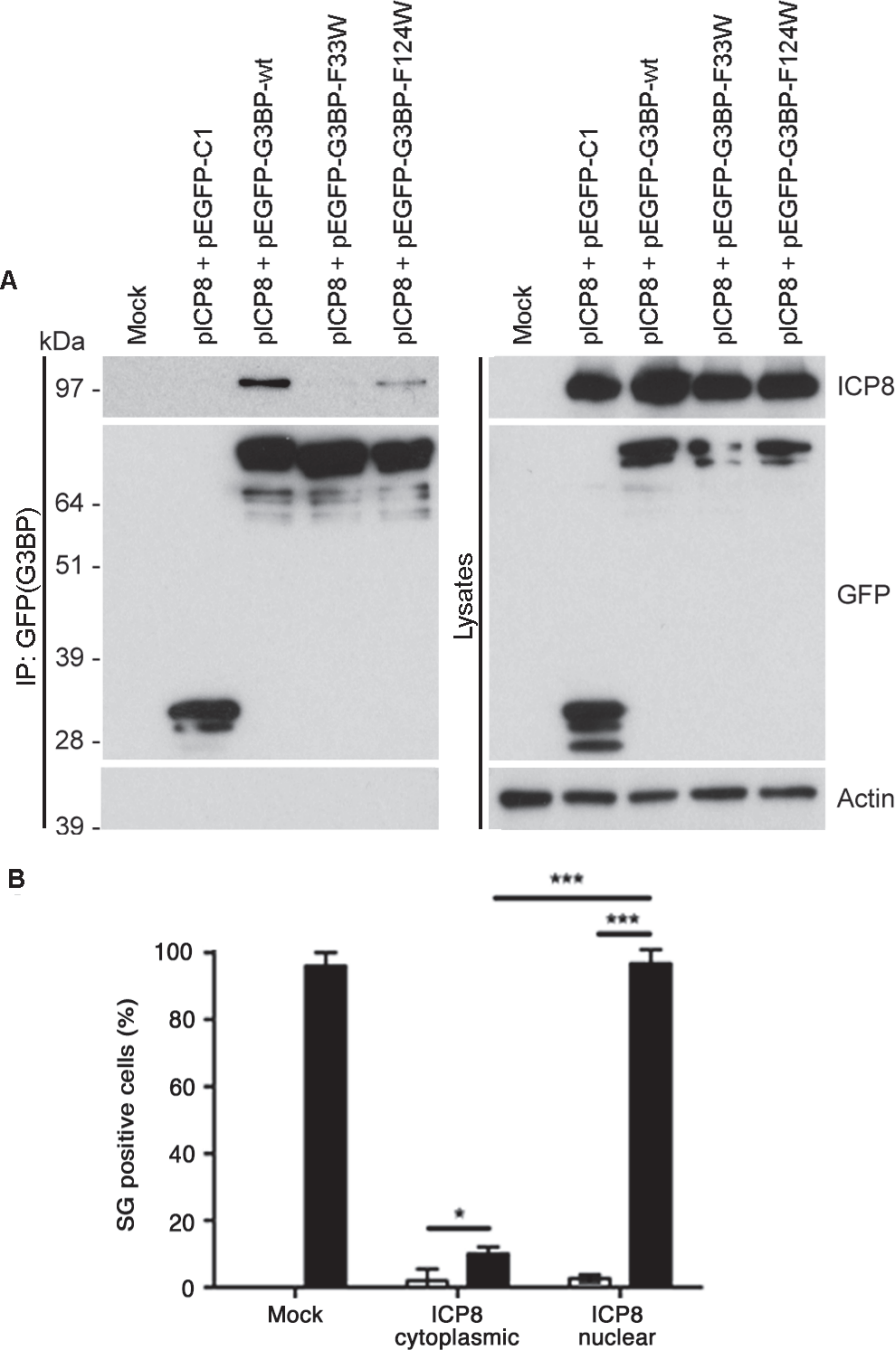
An FGDF motif in HSV-1 ICP8 binds G3BP and inhibits SGs. (A) HEK293T cells were mock transfected or cotransfected with pE29 (ICP8) and either pEGFP-C1, pEGFP-G3BP-wt, -F33W or -F124W. Cell lysates were prepared 24 h after transfection and immunoprecipitated with anti-GFP and separated by SDS–PAGE. Lysates and IPs were probed for ICP8, GFP or actin. (B) BHK cells were mock transfected or transfected with pE29 (ICP8). After 23 h the transfected cells were mock treated or treated with sodium arsenite for 1 h fixed and stained for ICP8, G3BP-1 and TIA1. ICP8+ cells were categorized as cytoplasmic or nuclear, and scored as SG+ based on G3BP1 and TIA1 colocalization. Open bars, mock treated; closed bars, sodium arsenite. Data are presented as mean +/- SD from three independent experiments in which 50 cells per treatment were counted. Unpaired Student’s t-test, * p < 0.05, *** p < 0.001.

Next, we asked whether the FGDF motif, mediating G3BP binding, is present in proteins other than SFV nsP3 and USP10. To identify such proteins, we searched the UniProtKB human and virus databases for all proteins containing FGxF motifs, where x = D, E or S, and also containing at least two acidic residues within the downstream 5 residues (as in both SFV nsP3 and USP10). Glutamic acid was permitted in the third position due to its chemical similarity to aspartic acid, while serine was permitted since Sindbis virus nsP3, shown by several researchers to bind G3BP-1 [25,26], contains a serine at that position. We identified 34 human (S1 Table) and 32 viral (S2 Table) sequences that meet these criteria. In one of those proteins, the infected cell protein (ICP)8 of herpes simplex virus (HSV)-1, the FGDF motif is located between residues 1144 and 1147 in the C-terminal region of the 1196 aa-long protein that is not predicted to adopt any specific secondary structure [34] and contains 3 acidic residues within the downstream 5 residues. This is reminiscent of the context of the FGDF motifs in Old World alphavirus nsP3, an otherwise quite different protein. ICP8 is a single stranded DNA binding protein that is expressed in the lytic cycle of herpes simplex viral replication. It is one of seven viral proteins that are necessary for viral DNA replication [35]. The FGDF motif in ICP8 is conserved in several HSV-1 strains and also in HSV-2 (S2 Table). To determine whether ICP8 binds G3BP-1, 293T cells were cotransfected with expression plasmids encoding HSV-1 ICP8 together with plasmids encoding EGFP-C1, EGFP-G3BP-wt, -F33W or -F124W. Lysates were immunoprecipitated with anti-GFP and immunoblotted for ICP8, GFP or actin (Fig. 7A). The results indicate that ICP8 indeed forms a complex with EGFP-G3BP-wt and EGFP-G3BP-F124W, but not with EGFP-C1 or EGFP-G3BP-F33W. These results are strikingly similar to the binding profiles of SFV nsP3 and USP10 (Fig. 6B and C), and strongly suggest that HSV-1 ICP8 forms a complex with G3BP-1 via binding of the FGDF motif at residues 1144–1147.

Since the binding of nsP3 and USP10 to G3BP inhibits SGs, we hypothesized that ICP8 binding might do the same. To investigate this, BHK cells were transfected with an ICP8 expression plasmid, stressed with sodium arsenite and stained for ICP8 as well as for G3BP-1 and TIA-1 to detect SGs. We observed that in 59% of the transfected cells, ICP8 accumulated mainly in the cytoplasm, while in the remaining 41%, ICP8 was localized in the nucleus. In neither case was there a detectable change in the localization of either G3BP-1 or TIA-1 (S8 Fig.), with these proteins exhibiting diffuse cytoplasmic or nuclear staining, respectively. After sodium arsenite stress however, only 10% of cells with predominantly cytoplasmic ICP8 contained TIA-1 and G3BP-1-positive SGs, while 96% of the cells with nuclear ICP8 and a similar proportion of mock-transfected cells had SGs (Fig. 7B). Representative images are provided in S8 Fig., showing that, when localized in the cytoplasm, ICP8 blocks the induction of SGs after sodium arsenite treatment. Due to the diffuse staining pattern of cytoplasmic ICP8, it is not possible to discern the proportion that is interacting with G3BP, but the inhibition of SG assembly was profound. Taken together with the results in Fig. 7A, this suggests that ICP8, like the cellular protein USP10 and SFV nsP3, interacts with G3BP via its FGDF-motif in a manner which blocks the formation of SGs

## Discussion

This work describes the FGDF motif, shared by proteins from at least two evolutionarily distant viruses and one cellular protein, that mediates strong binding to the multifunctional G3BP proteins. Among its many roles, G3BP is a critical determinant of SG assembly and is one of few proteins whose overexpression induces SGs [2,36]. We have shown that the binding of SFV nsP3, HSV-1 ICP8 and USP10 to G3BP via their FGDF motifs blocks SG formation (Figs. 2, 3D and 7B). The SG-nucleating function of G3BP requires both the RRM domain and the NTF2-like domain [2]. During stress, the RRM domain likely binds to translationally silent mRNAs or stalled 40S ribosomal subunits, and together with other RNA-binding proteins, targets them for SG inclusion. The SGs are then assembled via protein/protein interactions between SG-critical molecules. The NTF2-like domain, which binds the FGDF motifs, is involved in interactions with another critical regulator of SGs, caprin-1 [7]. It is therefore likely that the FGDF-mediated interactions inhibit or alter the ability of G3BP to form complexes with other proteins during the early stages of SG nucleation, resulting in an inhibition of SG formation. This work therefore reveals a mechanism by which USP10 acts as a negative regulator of SG formation. Furthermore, since G3BP appears to be targeted by many different viruses, its neutralization seems to be important for viral pathogenesis. Our work presents a common mechanism whereby Old World alphaviruses and at least two members of the alphaherpesviruses disrupt SG assembly.

Biophysical analyses demonstrated that one 25-mer peptide containing the two FGDF-motifs of SFV nsP3 binds two molecules of G3BP *in vitro*, suggesting that nsP3 of SFV and the other Old World alphaviruses binds and recruits two molecules of G3BP per nsP3 molecule (Fig. 4). We have previously shown that early in SFV infection, SGs are quickly disassembled in the vicinity of the newly established viral replication complexes [29]. The stoichiometry of the interaction may explain the rapidity of SG disassembly by nsP3, despite its relatively low expression compared to G3BP-targeting proteins of other lytic RNA viruses, such as the picornaviruses. It should be noted that a mutated SFV containing only one FGDF motif (SFV-Δ78) exhibited lower levels of G3BP binding and slower replication kinetics compared to wt SFV, but higher levels of G3BP binding and faster replication kinetics compared to SFV-Δ789, lacking both FGDF motifs [28]. Unlike other proteins with FGDF motifs such as USP10 and ICP8, the Old World alphavirus nsP3 has evolved to comprise two consecutive FGDF motifs to ensure rapid and efficient SG disassembly. This appears to be the main function of G3BP sequestration by nsP3’s FGDF motifs and represents an efficient evasion strategy, beneficial for virus replication.

Herpes simplex viruses block the induction of SGs via multiple mechanisms, highlighting the potent anti-viral effect of SGs. Although early events in viral infection activate PKR, the viral ICP34.5 protein promotes the protein phosphatase 1 (PP1)-mediated dephosphorylation of eIF2α and reactivation of translation [37]. HSV-1 mutants lacking the virion host shutoff (vhs) protein, an endoribonuclease that degrades cellular and viral mRNA, induce SGs late in infection [38,39]. This suggests that vhs may itself have a role in the inhibition of SGs or may alter expression of other SG-modulating viral gene products. HSV-2 also blocks the formation of SGs induced by sodium arsenite but not Pat A [40]. Here, we identify an FGDF motif at the C-termini of the HSV-1 and HSV-2 ICP8 proteins, and demonstrate that HSV-1 ICP8 binds G3BP and blocks SG formation (Fig. 7). Although ICP8 is a predominantly nuclear protein during HSV-1 infection, a sizeable fraction of the protein remains in the cytoplasm [41]. Functions of that fraction are not well described. Our results suggest that the cytoplasmic fraction of ICP8 inhibits SG assembly or other functions of G3BP. It remains to be determined if ICP8 contributes to the inhibition of SGs during HSV infection.

USP10 is a nucleocytoplasmic deubiquitinating enzyme (DUB), originally shown to be regulated by its binding partner G3BP [8]. USP10 deubiquitinates many different proteins of importance in several human diseases and is also a resident SG protein [16,22]. Here, we have shown that overexpression of EGFP-USP10, but not a mutant lacking an intact FGDF-motif, efficiently blocks the formation of SGs (Fig. 3). Interestingly, recent work has shown that the compound resveratrol can inhibit the interaction of G3BP with USP10 by binding to the NTF2-like domain of G3BP, thereby stimulating USP10 DUB activity and stabilization of p53 [17]. It appears therefore that the FGDF-mediated G3BP/USP10 complex is mutually inhibitory, with G3BP inhibiting the DUB activity of USP10 and USP10 inhibiting the SG nucleating function of G3BP. Elucidation of these inhibitory mechanism(s) will require further studies.

A molecular model reveals that the FGDF peptide binds tightly into a hydrophobic groove on the surface of the G3BP NTF2-like domain (Fig. 5). Mutagenesis analyses demonstrated that both phenylalanine residues and the glycine residue are required for binding, with a strong preference for aspartate in the third position.

Both phenylalanine side chains fit snugly within the binding site, the glycine is required for flexibility and the aspartate binds to the G3BP residue K123. Both phenylalanine and the glycine residues are fully conserved in most Old World alphavirus nsP3 sequences and in the USP10 proteins of all higher eukaryotes. The aspartate residue in the third position of the motif is conserved in many of the Old World alphavirus nsP3 sequences except that of Sindbis virus, in which it is a serine. A serine is also found in this position in the *Arabidopsis thaliana* USP10 gene, while this residue is a glutamate in avian and fish USP10 genes. We note the congruence of our biochemical, phylogenetic and structural analyses and propose that the core binding motif consists of FGxF, in which x can be aspartate, glutamate or serine. While mutation of the aspartate (D7) immediately downstream of the FGDF motif in SFV nsP3 had little effect on binding (Fig. 1B), it is notable that the FGDF motifs in SFV nsP3, USP10 and HSV-1 ICP8 are all followed by at least two acidic residues within the downstream four residues. In our molecular model, we observe that these residues are likely involved in interactions with the basic residues localized the N-terminus of G3BP, which is required for FGDF binding (Fig. 6E) and we propose that they constitute an important part of the motif that further stabilizes the complex. Using these criteria, we present a list of human and viral proteins that contain this motif, and therefore are candidate G3BP-binding proteins (S1 and S2 Tables).

In conclusion, our work describes a motif shared by three otherwise very different proteins, and potentially others, that mediates binding to G3BP and thereby inhibits SG formation. Our three-dimensional model provides a structural understanding of the G3BP/FGDF interaction and will form the basis for the design of pharmaceuticals to target this interaction with a therapeutic potential for a range of viral infections as well as cancers.

## Materials and Methods

### Plasmids, cell culture, and virus propagation

Expression vectors for EGFP-nsP3-31 [28], nsP3-BAP [27,42], G3BP-NTF2 [27], and HSV-1 ICP8 [43] were described previously. pEGFP-USP10_1–40_ wt sequences and corresponding alanine mutants were obtained from GeneArt, and ligated between the *BglII* and *EcoRI* sites of pEGFP-C1. Construction of the infectious clone pCMV-SFV-F3A: The PCR product derived from primers 1 and 2 (S3 Table) and the PCR product derived from primers 3 and 4 were fused by a one-step PCR in a molar ratio of 1:1. The DNA was denatured and annealed at 46˚C for 2 min. These partially double-stranded molecules were made fully double stranded by extension at 72˚C for 3 min. The fusion DNA was then amplified by using primer 1 and 4 for 25 cycles of PCR consisting of treatment at 95˚C for 30 s, 69˚C for 30 s, and 72˚C for 2 min, followed by a final extension at 72˚C for 5 min. The derived PCR product was purified and subcloned into pTZ57R/T plasmid (Thermo Scientific). The resulting pTZ57R/T-F3A plasmid was digested with XhoI and BglII and religated to the similar digested pCMV-SFV4 vector [44]. The presence of mutations was confirmed by sequencing. Construction of pEGFP-G3BP-F33W and -F124W: The PCR product derived from primers 5 and 6 (F33W) or primers 5 and 7 (F124W) and the product derived from primers 8 and 10 (F33W) or primers 9 and 10 (F124W) were fused by a one-step PCR in a molar ratio of 1:1. The DNA was denatured and annealed at 33˚C for 2 min. These partially double-stranded molecules were made fully double stranded by extension at 72˚C for 3 min. The fusion DNAs containing the F33W mutation or F124W mutation were then amplified by using primers 5 and 10 for 25 cycles of PCR consisting of treatment at 95˚C for 30 s, 56˚C for 30 s, and 72˚C for 2 min, followed by a final extension at 72˚C for 5 min. The derived PCR product was purified and subcloned into pTZ57R/T plasmid (Thermo Scientific). The resulting pTZ57R/T-G3BP1-F33W, -F124W plasmid was digested with BglII and EcoRI and religated to the similar digested pEGFP-C1-G3BP1 vector. The presence of mutations was confirmed by sequencing.

All cell lines were maintained as previously described [28,45,46]. Where indicated, cells were stressed by addition of sodium arsenite (0.5 mM) or pateamine A (100 nM) in complete medium for 60 min. Cells were transfected with Lipofectamine 2000 (Invitrogen) reagent according to the manufacturer’s instructions. Virus titration was performed by plaque assay, as previously described [28]

### Immunofluorescence, immunoprecipitations and immunoblotting

Immunofluorescence, immunoprecipitations and immunoblotting were performed as described previously (Panas et al., 2012). For details of all antibodies used, see S4 Table.

### Isothermal titration calorimetry (ITC)

His-tagged G3BP-NTF2 was expressed in *E*. *coli* BL21 T7 Express cells and purified using HisTrap columns (GE Healthcare). Before ITC, G3BP-NTF2 was eluted from a Superdex 75 HiLoad 16/60 (GE Healthcare) SEC column equilibrated in ITC-buffer (25 mM HEPES, 150 mM NaCl, 10 mM MgCl_2_, 10% glycerol, pH 7.5) at a retention volume of 60 mL. The peptides nsP3-25 and nsP3-25-mut were dissolved in ITC buffer at a concentration of 500 μM and dialyzed extensively against the same buffer. ITC measurements were performed using an ITC200 titration calorimeter (GE Healthcare). The cell temperature was set to 37°C, the reference power to 7 μCal/sec and the syringe stirring speed to 1000 rpm. G3BP-NTF2 was loaded into the cell. The peptides were titrated in 48 injections, each injection with a volume of 750 nL, a duration time of 1.5 sec and a waiting time between the injections of 150 sec. The first injection was performed using a volume of 300 nL, a duration time of 0.6 sec and a spacing time of 120 sec. Background measurements were performed with buffer injected into the protein solution, and peptide into the buffer solution. Data were analyzed using the Origin software as included in the instrument package.

### Analytical size exclusion chromatography (SEC) analysis

Before analysis, 40 μM G3BP-NTF2 and 400 μM nsP3-25 were mixed and incubated for 1h in SEC-buffer (25 mM HEPES, 300 mM NaCl, 5 mM MgCl_2_, 10% glycerol, pH 7). A sample volume of 100 μL was injected onto the Superdex 75 10/300 GL column equilibrated in SEC-buffer. G3BP-NTF2 alone was used a control. The apparent molecular weights of the eluted proteins were calculated from their retention volumes as described in the gel filtration LMW/HMW calibration kits assuming similar globular shapes for the analyzed proteins and calibration standards (GE Healthcare).

.

## Supporting Information

S1 Fig Mutagenesis of the G3BP-binding domain in SFV nsP3 reveals a core binding motif of FGDF. BHK cells were mock-transfected (M) or transfected with pEGFP-nsP3-31-wt, -T2A, -F3A, -G4A, -D5A, -F6A or -D7A or pEGFP-C1. Cell lysates were prepared 16 h after transfection and immunoprecipitated with anti-GFP, separated by SDS–PAGE and immunoblotted using an antibody which recognizes both isoforms of G3BP-2.
(TIF)

S2 Fig SFV-F3A is attenuated for growth in BHK cells. (A) BHK cells were infected with SFV-wt, SFV-F3A or SFV-∆789 at MOI 10. At 8 hpi, cell lysates were prepared, separated by SDS-PAGE and probed for nsP3 or actin. (B) BHK cells were infected with SFV-wt or SFV-F3A at MOI 10 (left) or MOI 0.1 (right). At 4, 8, 12, 24 and 36 hpi, supernatants were collected, and plaque-forming units (pfu) of SFV were quantified on BHK cells. Data are means of two independent experiments. Error bars indicate SD.
(TIF)

S3 Fig USP10 is not recruited to sites of nsP3 accumulation in SFV-infected cells. Stocks of SFV-b7EGFP recombinant virus particles were prepared as described previously [47] and used to infect MEFs at an MOI of 5. At 8 hpi, cells were fixed and stained for G3BP-1 (red) and USP10 (blue). Bar 20 μm.
(TIF)

S4 Fig USP10 binds G3BP via a conserved FGDF motif. BHK cells were mock-transfected (M) or transfected with pEGFP-C1 or pEGFP-USP10-40-wt, -F10A, -G11A, -D12A or -F13A. Cell lysates were prepared 16 h after transfection and immunoprecipitated with G3BP-1 antisera, separated by SDS–PAGE and probed for G3BP-1, GFP or actin.
(TIF)

S5 Fig USP10-wt, but not USP10-F10A blocks SG assembly upon sodium arsenite treatment. BHK cells were mock-transfected or transfected with pEGFP-C1, pEGFP-USP10-wt or -F10A. After 23 h the transfected cells were mock treated or treated with 0.5 mM sodium arsenite (SA) for 1 h, fixed and stained for G3BP-1 (red) and TIA-1 (blue). Bar 20 μm
(TIF)

S6 Fig Molecular model of G3BP/FGDF interaction. (A) Structure of G3BP-NTF2 with peptide SGFSF (green sticks), used as a template for model building (PDB: 4FCM). The peptide binds in the crevice on the surface of the protein. (B) Structure of G3BP-NTF2 with modelled peptide LTFGDFDE. Orientation of the complex is the same as in A.
(TIF)

S7 Fig G3BP-NTF2 alignments. (A) G3BP-1 in higher eukaryotes and (B) human G3BP-1 and -2. The numbering is based on the human sequences. Conserved residues are indicated in bold.
(TIF)

S8 Fig HSV-1 ICP8 blocks SG assembly upon sodium arsenite treatment. BHK cells were mock-transfected or transfected with pE29 (ICP8). After 23 h the transfected cells were mock treated or treated with sodium arsenite (SA) for 1 h fixed and stained for ICP8, G3BP-1 and TIA-1. Bar 20 μm.
(TIF)

S1 Table Human proteins containing FGxF motifs. The protein sequence database UniProtKB, filtered on the taxonomy homo sapiens, was scanned for the following motifs F-G-[DES]-F-[DE], F-G-[DES]-F-x-[DE], F-G-[DES]-F-x-x-[DE], F-G-[DES]-F-x-x-x[DE], F-G-[DES]-F-x-x-x-x-[DE]. Table is grouped according to FGDF, FGEF and FGSF motifs and within the groups sorted according to gene name.
(PDF)

S2 Table Viral proteins containing FGxF motifs. The protein sequence database UniProtKB, filtered on the taxonomy viruses, was scanned for the following motifs F-G-[DES]-F-[DE], F-G-[DES]-F-x-[DE], F-G-[DES]-F-x-x-[DE], F-G-[DES]-F-x-x-x[DE], F-G-[DES]-F-x-x-x-x-[DE].Table is grouped according to FGDF, FGEF and FGSF motifs and within the groups sorted according to gene name. The FGxF motif in the Togaviridae family can be found in the non-structural protein 3 (nsP3).
(PDF)

S3 Table List of primers. Restriction sites are underlined. Overlapping sequences are shown in italic and nucleotides encoding amino acid substitutions are shown in bold.
(PDF)

S4 Table List of antibodies. Antigen, species, application and source of all antibodies used in the study.
[48]
(PDF)

S1 Text Supporting Materials and Methods. (PDF)
