# Supplementary figures and images for: Viral and Cellular Proteins Containing FGDF Motifs Bind G3BP to Block Stress Granule Formation
Source: PLoS Pathog. 2015 Feb 6;11(2):e1004659. doi: 10.1371/journal.ppat.1004659 (PMC4450067; doi:10.1371/journal.ppat.1004659)

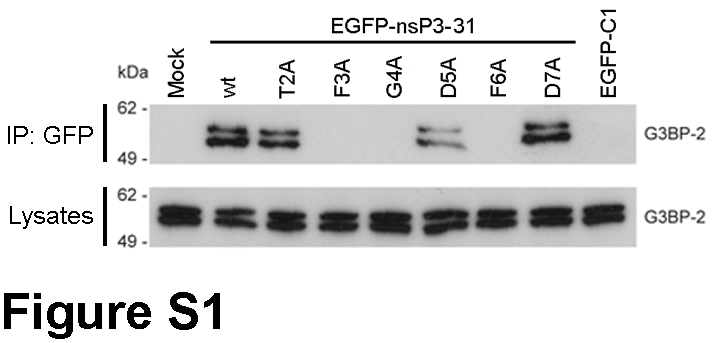

Supplement: S1 Fig — BHK cells were mock-transfected (M) or transfected with pEGFP-nsP3-31-wt, -T2A, -F3A, -G4A, -D5A, -F6A or -D7A or pEGFP-C1. Cell lysates were prepared 16 h after transfection and immunoprecipitated with anti-GFP, separated by SDS–PAGE and immunoblotted using an antibody which recognizes both isoforms of G3BP-2. (TIF) [file ppat.1004659.s001.tif]

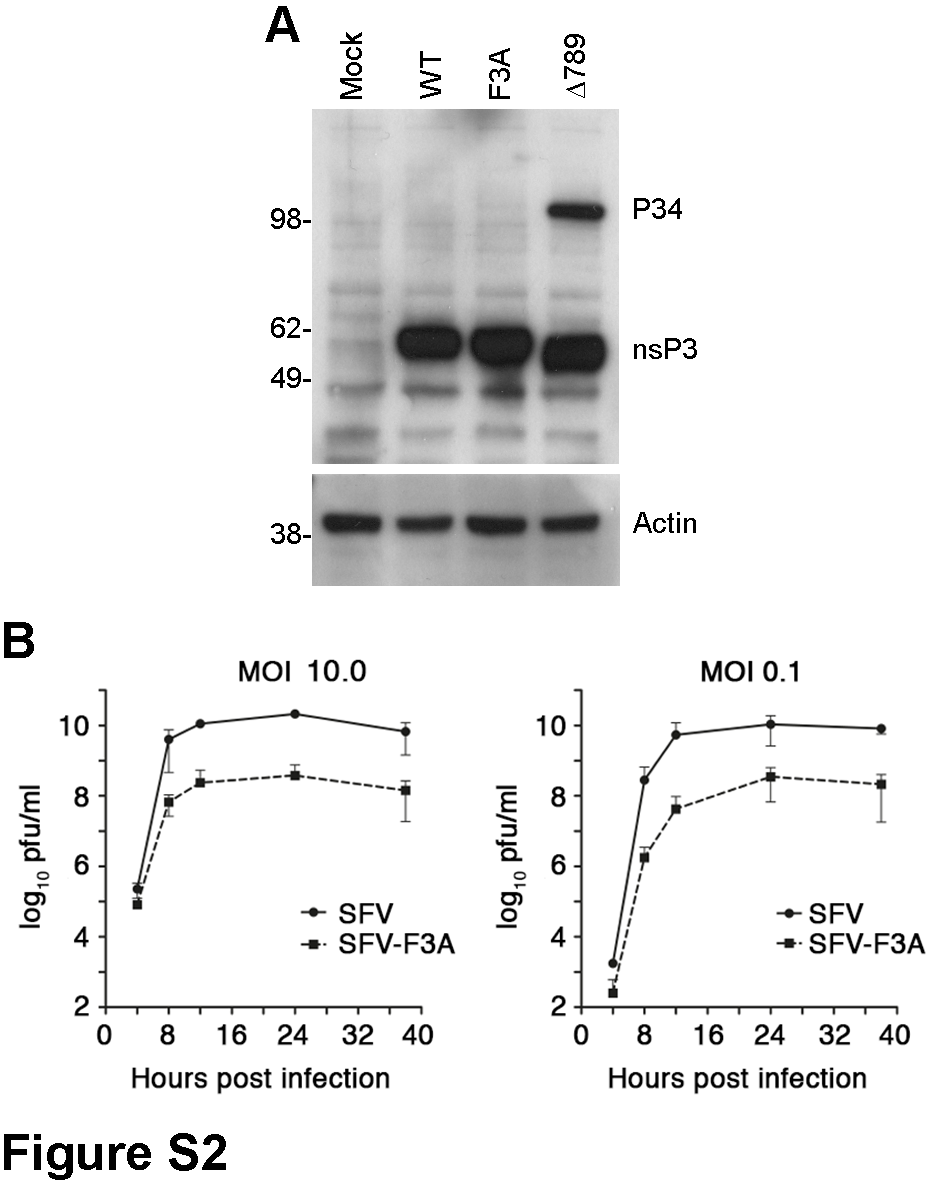

Supplement: S2 Fig — (A) BHK cells were infected with SFV-wt, SFV-F3A or SFV-∆789 at MOI 10. At 8 hpi, cell lysates were prepared, separated by SDS-PAGE and probed for nsP3 or actin. (B) BHK cells were infected with SFV-wt or SFV-F3A at MOI 10 (left) or MOI 0.1 (right). At 4, 8, 12, 24 and 36 hpi, supernatants were collected, and plaque-forming units (pfu) of SFV were quantified on BHK cells. Data are means of two independent experiments. Error bars indicate SD. (TIF) [file ppat.1004659.s002.tif]

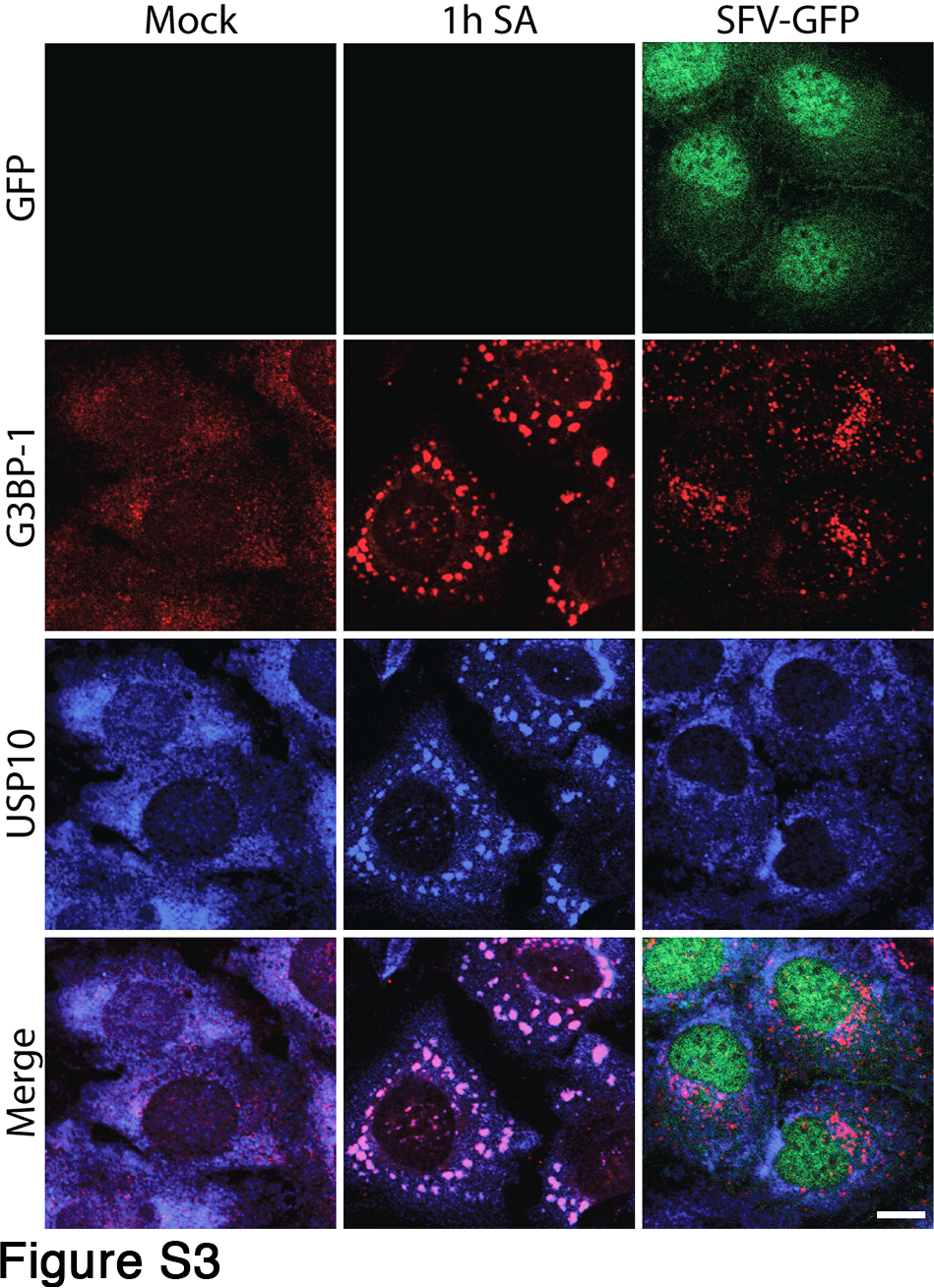

Supplement: S3 Fig — Stocks of SFV-b7EGFP recombinant virus particles were prepared as described previously [47] and used to infect MEFs at an MOI of 5. At 8 hpi, cells were fixed and stained for G3BP-1 (red) and USP10 (blue). Bar 20 μm. (TIF) [file ppat.1004659.s003.tif]

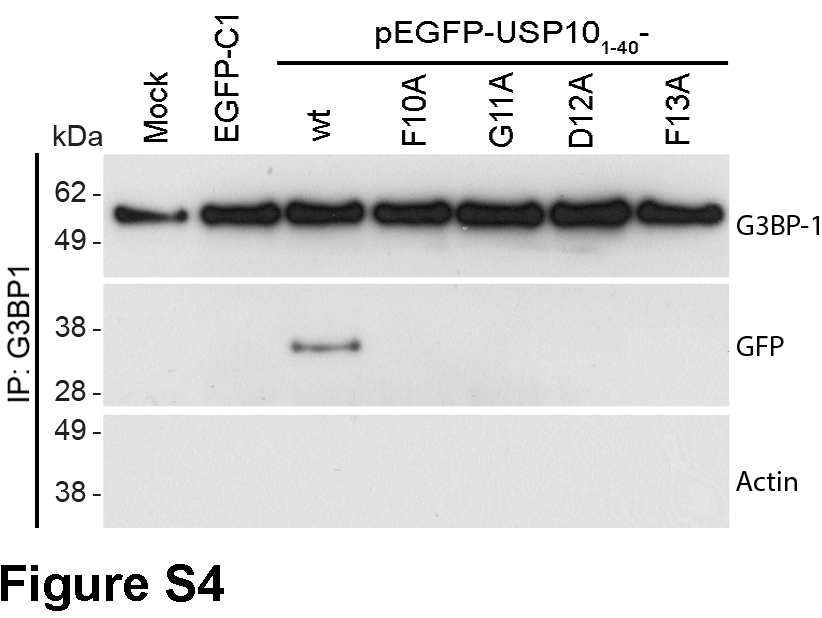

Supplement: S4 Fig — BHK cells were mock-transfected (M) or transfected with pEGFP-C1 or pEGFP-USP10-40-wt, -F10A, -G11A, -D12A or -F13A. Cell lysates were prepared 16 h after transfection and immunoprecipitated with G3BP-1 antisera, separated by SDS–PAGE and probed for G3BP-1, GFP or actin. (TIF) [file ppat.1004659.s004.tif]

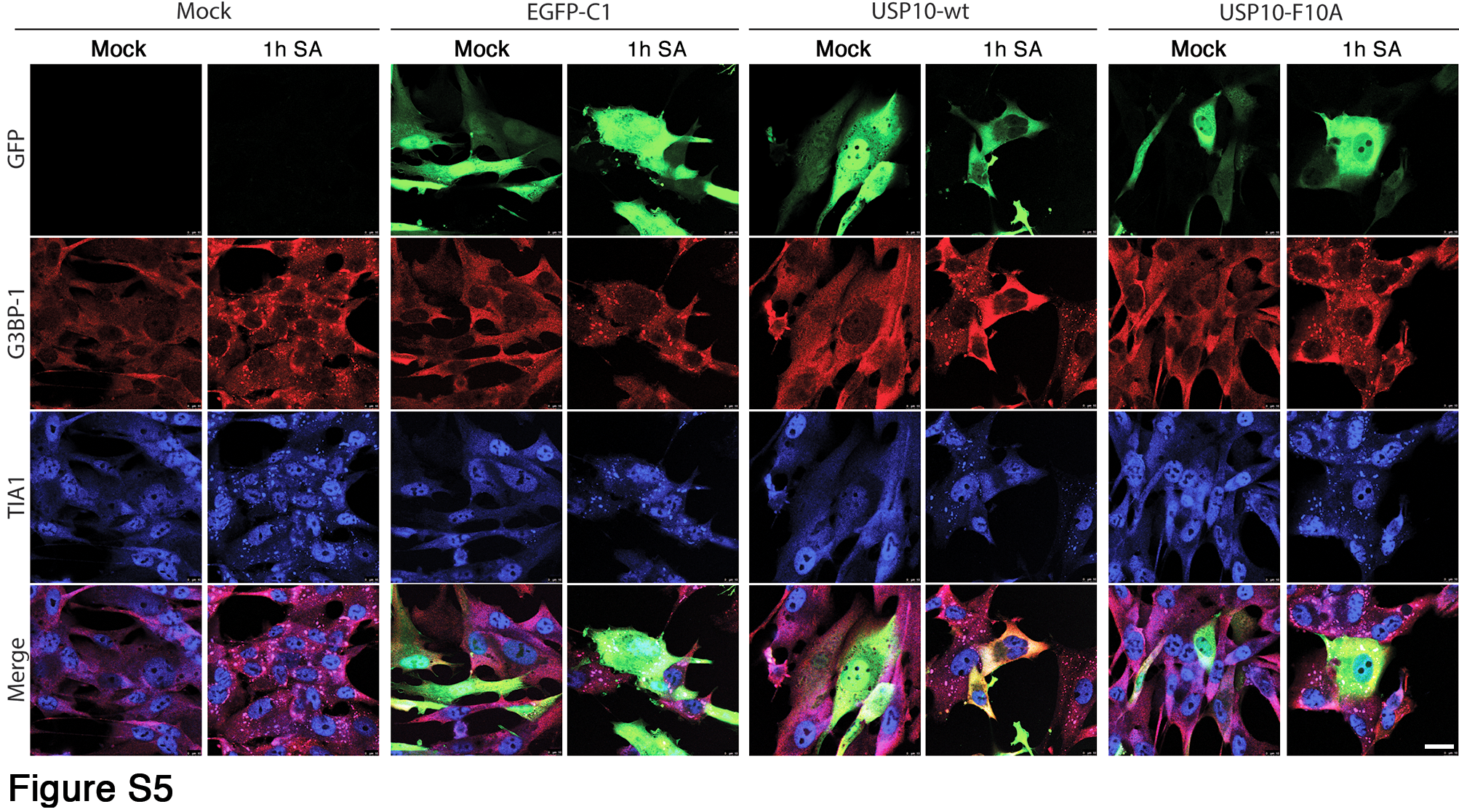

Supplement: S5 Fig — BHK cells were mock-transfected or transfected with pEGFP-C1, pEGFP-USP10-wt or -F10A. After 23 h the transfected cells were mock treated or treated with 0.5 mM sodium arsenite (SA) for 1 h, fixed and stained for G3BP-1 (red) and TIA-1 (blue). Bar 20 μm (TIF) [file ppat.1004659.s005.tif]

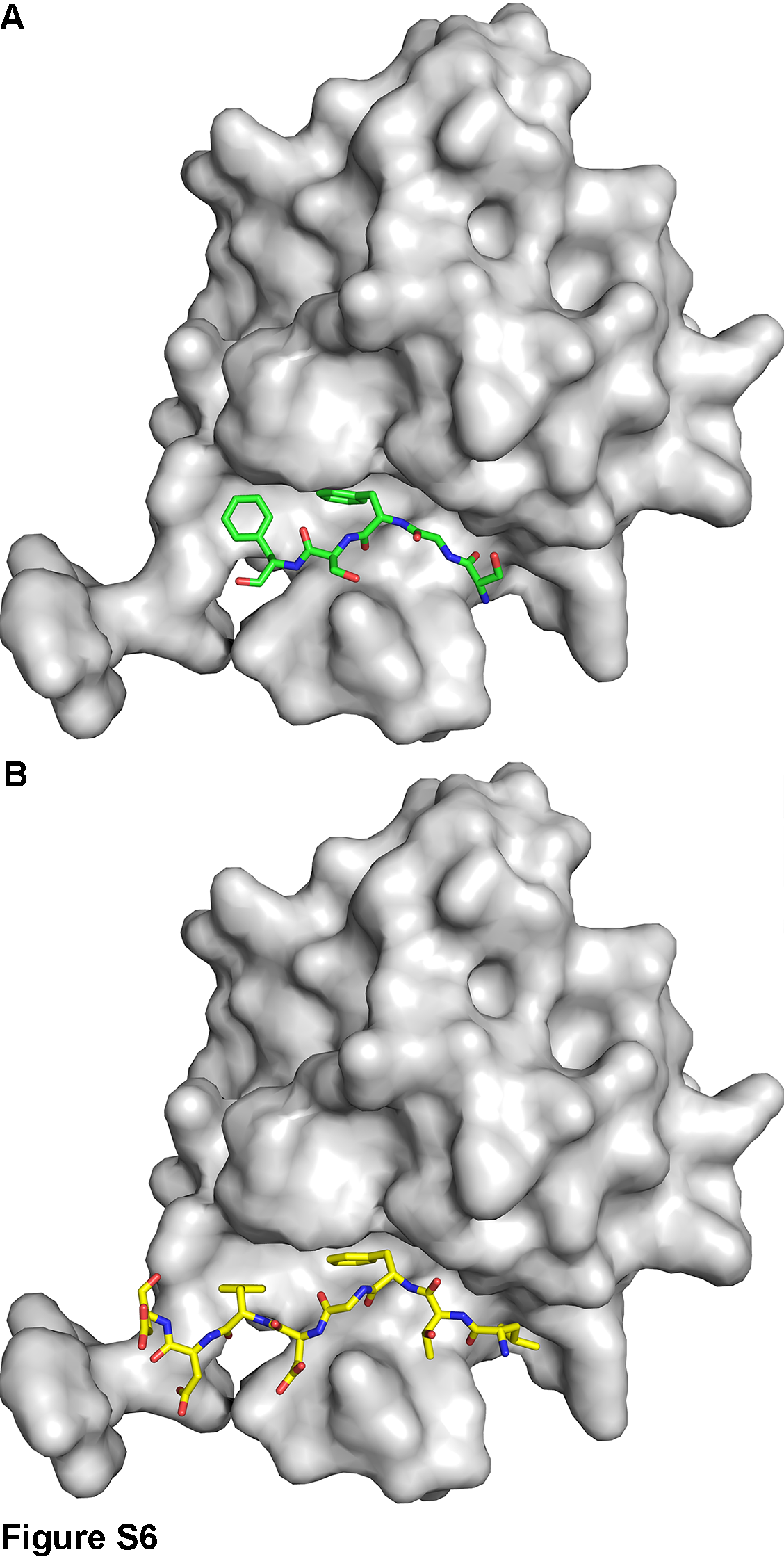

Supplement: S6 Fig — (A) Structure of G3BP-NTF2 with peptide SGFSF (green sticks), used as a template for model building (PDB: 4FCM). The peptide binds in the crevice on the surface of the protein. (B) Structure of G3BP-NTF2 with modelled peptide LTFGDFDE. Orientation of the complex is the same as in A. (TIF) [file ppat.1004659.s006.tif]

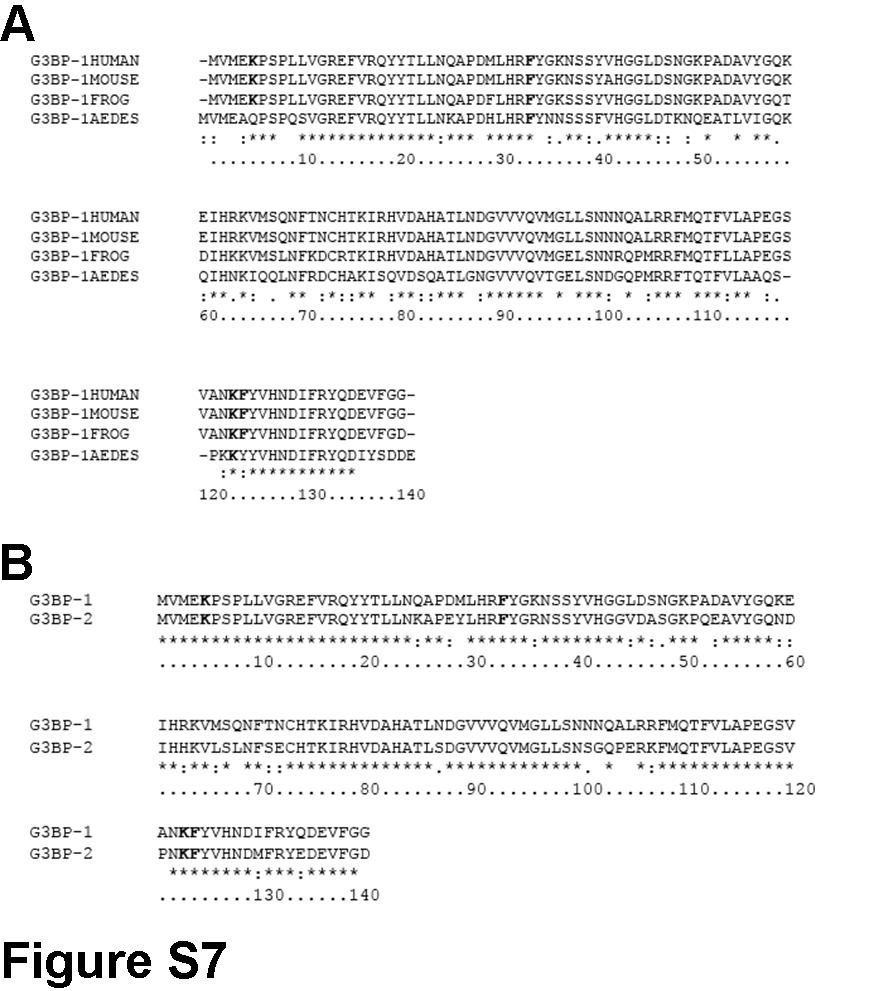

Supplement: S7 Fig — (A) G3BP-1 in higher eukaryotes and (B) human G3BP-1 and -2. The numbering is based on the human sequences. Conserved residues are indicated in bold. (TIF) [file ppat.1004659.s007.tif]

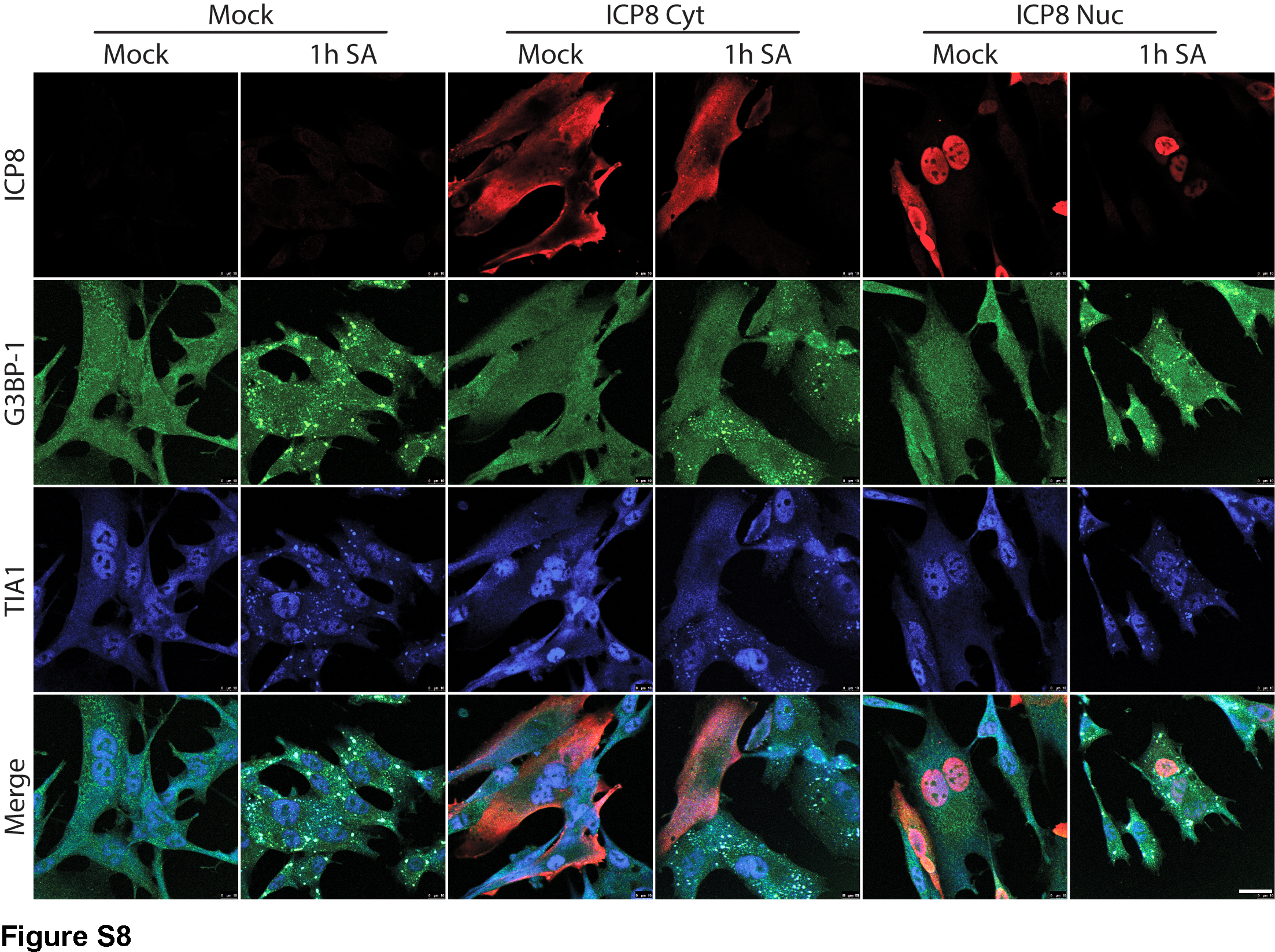

Supplement: S8 Fig — BHK cells were mock-transfected or transfected with pE29 (ICP8). After 23 h the transfected cells were mock treated or treated with sodium arsenite (SA) for 1 h fixed and stained for ICP8, G3BP-1 and TIA-1. Bar 20 μm. (TIF) [file ppat.1004659.s008.tif]
