# Supplementary material for: Viral and Cellular Proteins Containing FGDF Motifs Bind G3BP to Block Stress Granule Formation
Source: PLoS Pathog. 2015 Feb 6;11(2):e1004659. doi: 10.1371/journal.ppat.1004659 (PMC4450067; doi:10.1371/journal.ppat.1004659)
Supplement: S1 Table — The protein sequence database UniProtKB, filtered on the taxonomy homo sapiens, was scanned for the following motifs F-G-[DES]-F-[DE], F-G-[DES]-F-x-[DE], F-G-[DES]-F-x-x-[DE], F-G-[DES]-F-x-x-x[DE], F-G-[DES]-F-x-x-x-x-[DE]. Table is grouped according to FGDF, FGEF and FGSF motifs and within the groups sorted according to gene name. (PDF) [file ppat.1004659.s009.pdf]

Gene Name	Protein Name	Length (aa)	Location	Pattern	UniProt ID
			**FGDF**		
ASTN2	Astrotactin-2.	1339	1247 - 1250	NSHYEK**FGDF**VWRS**EDE**	O75129
BTAF1	TATA-binding protein-associated factor 172	1849	355 - 358	VFALDR**FGDF**VS**DE**VVA	O14981
CS044	Uncharacterized protein C19orf44.	657	15 - 18	RPMRDV**FGDF**S**D**VSL**ED**	Q9H6X5
DCBD2	Discoidin, CUB and LCCL domain-containing protein 2	775	115 - 118	ERVRIK**FGDFD**I**ED**S**D**S	Q96PD2
DHX37	Probable ATP-dependent RNA helicase DHX37	1157	689 - 692	LYSSAV**FGDFE**QFPPP**E**	Q8IY37
DPOLB	DNA polymerase beta	335	143 - 146	RIGLKY**FGDFE**KRIPR**E**	P06746
FRPD4	FERM and PDZ domain-containing protein 4	1322	655 - 658	AKVSFI**FGDF**AL**DD**GIS	Q14CM0
PPARG	Peroxisome proliferator-activated receptor gamma	505	388 - 391	KSLRKP**FGDF**M**E**PKF**E**F	P37231
PTN21	Tyrosine-protein phosphatase non-receptor type 21	1174	152 - 155	LAVQAD**FGDFD**QY**E**SQ**D**	Q16825
RAD21	Double-strand-break repair protein rad21 homolog	631	160 - 163	ILQEND**FGDF**GM**DD**R**E**I	O60216
TTC23	Tetratricopeptide repeat protein 23	447	348 - 351	EAKVEA**FGDF**SP**E**VA**E**T	Q5W5X9
USP10	Ubiquitin carboxyl-terminal hydrolase 10	798	10 - 13	HSPQYI**FGDF**SP**DE**FNQ	Q14694
			**FGEF**		
ASTN1	Astrotactin-1	1302	1210 - 1213	NQHYES**FGEF**TWRC**EDE**	O14525
CLCN6	Chloride transport protein 6	869	314 - 317	LPGLLN**FGEF**KCS**D**S**D**K	P51797
EBLN1	Endogenous Bornavirus-like nucleoprotein 1	366	211 - 214	LMFTFL**FGEFE**SPAC**E**L	P0CF75
ORC4	Origin recognition complex subunit 4	436	129 - 132	EKIREY**FGEF**G**E**I**E**AI**E**	O43929
ROAA	Heterogeneous nuclear ribonucleoprotein A/B	332	174 - 177	EKIREY**FGEF**G**E**I**E**AI**E**	Q99729
			**FGSF**		
FMN1	Formin-1	1419	250 - 253	PDTDLG**FGSFE**TAFK**D**T	Q68DA7
ITB5	Integrin beta-5	799	179 - 182	SNFRLG**FGSF**V**D**K**D**ISP	P18084
KI2L1	Killer cell immunoglobulin-like receptor 2DL1	348	199 - 203	GGTYRC**FGSF**H**D**SPY**E**W	P43626
KI2L2	Killer cell immunoglobulin-like receptor 2DL2	348	199 - 202	GGTYRC**FGSF**R**D**SPY**E**W	P43627
KI2S3	Killer cell immunoglobulin-like receptor 2DS3	304	199 - 203	GGTYRC**FGSF**H**D**SPY**E**W	Q14952
KI3P1	Putative killer cell immunoglobulin-like receptor like protein KIR3DP1	328	294 - 297	GGTYRC**FGSF**R**D**SPY**E**W	A8MWS1
LIRA1	Leukocyte immunoglobulin-like receptor subfamily A member	489	151 - 154	CVSQVA**FGSF**ILCK**E**G**E**	O75019
LONP2	Lon protease homolog 2, peroxisomal	852	717 - 720	YQLTNA**FGSFD**LL**D**NT**D**	Q86WA8
LR14B	Leucine-rich repeat-containing protein 14B	514	477 - 480	QVSTPL**FGSFD**P**D**IQ**E**T	A6NHZ5
MAP1B	Microtubule-associated protein 1B	2468	1510 - 1513	QIDVSQ**FGSF**K**ED**TKMS	P46821
N4BP2	NEDD4-binding protein 2	1770	1099 - 1102	NILCKL**FGSF**SL**E**ALK**D**	Q86UW6
PO4F3	POU domain, class 4, transcription factor 3	338	46 - 49	QLQGNI**FGSFDE**SLLAR	Q15319
RGS1	Regulator of G-protein signaling 1	209	99 - 102	QTGQNV**FGSF**LKS**E**FS**E**	Q08116
SELO	Selenoprotein O	669	254 - 257	ASTFIR**FGSFE**IFKSA**D**	Q9BVL4
SODM	Superoxide dismutase [Mn], mitochondrial	222	125 - 128	EAIKRD**FGSFD**KFK**E**KL	P04179
UNC80	Protein unc-80 homolog	3258	494 - 497	TRSTFS**FGSF**SGLG**ED**R	Q8N2C7
VILI	Villin-1	827	37 - 40	PVPSST**FGSF**F**D**G**D**CYI	P09327

Table S1
