# Supplementary material for: Viral and Cellular Proteins Containing FGDF Motifs Bind G3BP to Block Stress Granule Formation
Source: PLoS Pathog. 2015 Feb 6;11(2):e1004659. doi: 10.1371/journal.ppat.1004659 (PMC4450067; doi:10.1371/journal.ppat.1004659)
Supplement: S2 Table — The protein sequence database UniProtKB, filtered on the taxonomy viruses, was scanned for the following motifs F-G-[DES]-F-[DE], F-G-[DES]-F-x-[DE], F-G-[DES]-F-x-x-[DE], F-G-[DES]-F-x-x-x[DE], F-G-[DES]-F-x-x-x-x-[DE].Table is grouped according to FGDF, FGEF and FGSF motifs and within the groups sorted according to gene name. The FGxF motif in the Togaviridae family can be found in the non-structural protein 3 (nsP3). (PDF) [file ppat.1004659.s010.pdf]

Gene Name	Protein Name	Length (aa)	Virus	Virus family	Genome	Location	Pattern	UniProt ID
				**FGDF**				
DNBI_HHV11	Major DNA-binding protein	1196	HHV-1 Human herpes simplex virus 1	Herpesviridae	dsDNA	1144 - 1147	AGEVFNFGDFGCEDDNA	P04296
DNBI_HHV1F	Major DNA-binding protein	1196	HHV-1 Human herpes simplex virus 1 (strain F)	Herpesviridae	dsDNA	1144 - 1147	AGEVFNFGDFGCEDDNA	P17469
DNBI_HHV1K	Major DNA-binding protein	1196	HHV-1 Human herpes simplex virus 1 (strain KOS)	Herpesviridae	dsDNA	1144 - 1152	AGEVFNFGDFGCEDDNA	P17470
DNBI_HHV2	Major DNA-binding protein	1197	HHV-2 Human herpes simplex virus 2	Herpesviridae	dsDNA	1144 - 1147	AGEVFNFGDFGDADDHA	P36384
DNBI_HHV2H	Major DNA-binding protein	1196	HHV-2 Human herpes simplex virus 2 (strain HG52)	Herpesviridae	dsDNA	1144 - 1147	AGEVFNFGDFGDEDDHA	P89452
HELI_EBVB9	DNA replication helicase	809	Epstein-Barr virus (strain B95-8)	Herpesviridae	dsDNA	479 - 482	CGYTGTFGDFMDTLEAD	P03214
POLN_AURAV	Non-structural polyprotein	2498	Aura virus	Togaviridae	+ssRNA	1845 - 1848<br>1865 - 1868	LSMPITFGDFAEGELDR<br>PSPTPTFGDFSQEEMDR	Q86924
POLN_BFV	Non-structural polyprotein	2410	Barmah Forest virus	Togaviridae	+ssRNA	1760 - 1763<br>1778 - 1781	MHPGFTFGDFGEHEVEE<br>TASPLTFGDFAEGEIQG	P87515
POLN_CHIK3	Non-structural polyprotein	2474	Chikungunya virus (strain 37997)	Togaviridae	+ssRNA	1812 - 1815<br>1830 - 1833	ETFPITFGDFDEGEIES<br>SSELLTFGDFSPGEVDD	Q5XXP4
POLN_MAYAB	Non-structural polyprotein	2436	Mayaro virus strain	Togaviridae	+ssRNA	1804 - 1807	EESDITFGDFSASEWET	Q8QZ73
POLN_MIDDV	Non-structural polyprotein	994	Middelburg virus	Togaviridae	+ssRNA	364 - 367	INDDLTFGDFGAGEFER	P03318
POLN_ONNVG	Non-structural polyprotein	2514	O'nyong-nyong virus (strain Gulu)	Togaviridae	+ssRNA	1852 - 1855<br>1870 - 1873	TLEPITFGDFAEGEIDN<br>LTGALTFGDFEPGEVEE	P13886
POLN_RRVN	Non-structural polyprotein	2479	Ross river virus (strain NB5092)	Togaviridae	+ssRNA	1843 - 1846	EDVDIQFGDFETPDKIQ	P13887
POLN_SFV	Non-structural polyprotein	2432	Semliki Forest virus	Togaviridae	+ssRNA	1787 - 1790<br>1804 - 1807	NKLPLTFGDFDEHEVDA<br>LASGITFGDFDDVLRLG	P08411
THYX_BPML5	Probable thymidylate synthase	243	Mycobacterium phage L5	Siphoviridae	dsDNA	36 - 39	KDEDPYFGDFDADELAE	Q05259
VP12_BPPRD	Single-stranded DNA-binding protein	160	Enterobacteria phage PRD1	Tectiviridae	dsDNA	44 - 47	KVGQSTFGDFIKFEGEF	P17637
YL032_MIMIV	Putative KilA-N domain-containing protein L32	372	Acanthamoeba polyphaga mimivirus	Mimiviridae	dsDNA	157 - 160	KFAIGKFGDFEVIINRD	Q5UPA5

				**FGEF**				
MREP_SCSVF	Master replication protein	286	Subterranean clover stunt virus (strain F)	Nanoviridae	ssDNA	93 - 97	VEGPWE**FGEF**K**E**VL**ED**K	Q9ICP7
POLG_BVY3	Genome polyprotein	3491	Blackberry virus Y (isolate Blackberry plant/USA Arkansas/C3ARK/2005)	Potyvirdae	+ssRNA	328 - 331	TKTTIN**FGEF****E**V**D**GY	A0AUJ5
RDRP_NODAM	RNA-directed RNA polymerase	1043	Nodamura virus (strain Mag115)	Nodaviridae	+ssRNA	239 - 242	VWDWCA**FGEF**I**E**TR**D**AS	Q9IMM4
RDRP_WCCVB	RNA-directed RNA polymerase	616	White clover cryptic virus 1 (isolate Boccardo/2004)	Partitiviridae	dsRNA	152 - 155	LSQRKT**FGEF**IRMH**E**Y**E**	Q64FP0
				**FGSF**				
COAT_BPAR1	Major capsid protein	521	Enterobacteria phage AR1	Myoviridae	dsDNA	59 - 62	EKIAQA**FGSF**LT**E**A**E**IG	Q9ZXI0
COAT_BPT4	Major capsid protein	521	Enterobacteria phage T4	Myoviridae	dsDNA	59 - 62	EKIAQA**FGSF**LT**E**A**E**IG	P04535
COAT_BPT6	Major capsid protein	502	Enterobacteria phage T6	Myoviridae	dsDNA	59 - 62	EKIAQA**FGSF**LT**E**A**E**IG	Q38055
ETF2_MCV1	Early transcription factor 82 kDa subunit	707	Molluscum contagiosum virus subtype 1	Poxviridae	dsDNA	142 - 145	DTPVPE**FGSF****EE**V**D**ANI	Q98277
MVP_LMV0	P3N-PIPO polyprotein	1152	Lettuce mosaic virus (strain 0 / isolate French)	Potyvirdae	+ssRNA	131 - 134	KCRGLQ**FGSF**T**E**L**E**TS**E**	P0CJ97
MVP_LMVE	P3N-PIPO polyprotein	1152	Lettuce mosaic virus (strain E)	Potyvirdae	+ssRNA	131 - 134	KCRGLQ**FGSF**T**E**L**E**TS**E**	P0CW79
POL1_CRLVP	RNA1 polyprotein	2250	Cherry rasp leaf virus (isolate Potato/United States)	Secoviridae	+ssRNA	115 - 118	ASGPCF**FGSF**GPF**EE**YY	Q6EWG9
POL1_CRLVP	RNA1 polyprotein	2250	Cherry rasp leaf virus (isolate Potato/United States)	Secoviridae	+ssRNA	115 - 118	ASGPCF**FGSF**GPF**EE**YY	Q6EWG9
POLA_CHPVU	Polyprotein p69	622	Cryphonectria hypovirus 1 (strain Euro7)	Hypoviridae	dsRNA	200 - 203	WVGRRS**FGSF**QI**EE**STV	Q9YTU3
POLG_MCFA	Genome polyprotein	3341	Mosquito cell fusing agent CFA flavivirus	Flaviviridae	+ssRNA	2749 - 2752	YKSFKY**FGSF**VT**DD**VKV	P33515
POLN_SINDV	Non-structural polyprotein	2512	Sindbis virus	Togaviridae	+ssRNA	1837 - 1840	TDVPMS**FGSF**S**D**G**E**I**DE**<br>ESEPVL**FGSF****E**PG**E**VNS	P03317

Table S2
