# Supplementary material for: Viral and Cellular Proteins Containing FGDF Motifs Bind G3BP to Block Stress Granule Formation
Source: PLoS Pathog. 2015 Feb 6;11(2):e1004659. doi: 10.1371/journal.ppat.1004659 (PMC4450067; doi:10.1371/journal.ppat.1004659)
Supplement: S3 Table — Restriction sites are underlined. Overlapping sequences are shown in italic and nucleotides encoding amino acid substitutions are shown in bold. (PDF) [file ppat.1004659.s011.pdf]

**Name**	**Sequence 5´→ 3´**	**Features**
Primer 1	CCGCAGACCATGTGGACCTCGAGAACCCG	XhoI restriction site
Primer 2	*CGCATCGACCT*CGTGCTCGTCAAAGTCGCC**CGC**CGTCAAAG	Ala codon pos 444, Overlap primer 3 (12bp)
Primer 3	*GAGGTCGATGCG*TTGGCCTCCGGGATTACT**GCG**GGAGACTT	Overlap primer 2 (12 bp), Ala codon pos. 460
Primer 4	GTCCAGCAGGTACTGATCCACCCCTAGATCTTCGAGG	BglII restriction site
Primer 5	GACTCAGATCTATGGTGATGGAG	BglII restriction site
Primer 6	*GAGTTCTTTCC*ATA**CCA**TCTATGCAGCAT	Trp codon pos 33
Primer 7	*TCATTGTGAAC*ATA**CCA**TTTATTTGCAAC	Trp codon pos 124
Primer 8	*GGAAAGAACTC*TTCTTATGTCCATGG	11 bp overlap with primer 6
Primer 9	*GTTCACAATGA*TATCTTCAGATACCAAGA	11 bp overlap with primer 7
Primer 10	CGACTGCAGAATTCTTACTGC	EcoRI restriction site

Table S3
