# Supplementary material for: Viral and Cellular Proteins Containing FGDF Motifs Bind G3BP to Block Stress Granule Formation
Source: PLoS Pathog. 2015 Feb 6;11(2):e1004659. doi: 10.1371/journal.ppat.1004659 (PMC4450067; doi:10.1371/journal.ppat.1004659)
Supplement: S4 Table — Antigen, species, application and source of all antibodies used in the study. [48] (PDF) [file ppat.1004659.s012.pdf]

**Antigen**	**Species**	**Application**	**Source**
G3BP-1	rabbit	IF, WB	Aviva Systems
G3BP-1	mouse	IP, IF	BD Transduction
G3BP-2	rabbit	WB	Assay Biotech
nsP3	rabbit	IF, WB	(Tamberg et al., 2007)
USP10	rabbit	IF, WB	Abcam
ICP8	rabbit	IF, WB	Per Elias
GFP	rabbit	WB	Abcam
GFP	mouse	IP	Abcam
Streptavidin-HRP	rabbit	WB	Amersham
TIA-1	goat	IF	Santa Cruz
Actin	goat	WB	Santa Cruz

Table S4
