# Supplementary material for: Viral and Cellular Proteins Containing FGDF Motifs Bind G3BP to Block Stress Granule Formation
Source: PLoS Pathog. 2015 Feb 6;11(2):e1004659. doi: 10.1371/journal.ppat.1004659 (PMC4450067; doi:10.1371/journal.ppat.1004659)
Supplement: S1 Text — (PDF) [file ppat.1004659.s013.pdf]

Supporting Materials and Methods

Plasmids construction

Construction of the infectious clone pCMV-SFV-F3A: The PCR product derived from primers 1 and 2 (Table S3) and the PCR product derived from primers 3 and 4 were fused by a one-step PCR in a molar ratio of 1:1. The DNA was denatured and annealed at 46˚C for 2 min. These partially double-stranded molecules were made fully double stranded by extension at 72˚C for 3 min. The fusion DNA was then amplified by using primer 1 and 4 for 25 cycles of PCR consisting of treatment at 95˚C for 30 s, 69˚C for 30 s, and 72˚C for 2 min, followed by a final extension at 72˚C for 5 min. The derived PCR product was purified and subcloned into pTZ57R/T plasmid (Thermo Scientific). The resulting pTZ57R/T-F3A plasmid was digested with XhoI and BglII and religated to the similar digested pCMV-SFV4 vector (Ulper et al., 2008). The presence of mutations was confirmed by sequencing.

Construction of pEGFP-G3BP-F33W and -F124W: The PCR product derived from primers 5 and 6 (F33W) or primers 5 and 7 (F124W) and the product derived from primers 8 and 10 (F33W) or primers 9 and 10 (F124W) were fused by a one-step PCR in a molar ratio of 1:1. The DNA was denatured and annealed at 33˚C for 2 min. These partially double-stranded molecules were made fully double stranded by extension at 72˚C for 3 min. The fusion DNAs containing the F33W mutation or F124W mutation were then amplified by using primers 5 and 10 for 25 cycles of PCR consisting of treatment at 95˚C for 30 s, 56˚C for 30 s, and 72˚C for 2 min, followed by a final extension at 72˚C for 5 min. The derived PCR product was purified and subcloned into pTZ57R/T plasmid (Thermo Scientific). The resulting pTZ57R/T-G3BP1-F33W, -F124W plasmid was digested with BglII and EcoRI and religated to the similar digested pEGFP-C1-G3BP1 vector. The presence of mutations was confirmed by sequencing.

Supporting References

Ulper, L., Sarand, I., Rausalu, K., *et al.* (2008). Construction, properties, and potential application of infectious plasmids containing Semliki Forest virus full-length cDNA with an inserted intron. J Virol Methods *148*, 265-270.
